# Ringiculid bubble snails recovered as the sister group to sea slugs (Nudipleura)

**DOI:** 10.1038/srep30908

**Published:** 2016-08-08

**Authors:** Yasunori Kano, Bastian Brenzinger, Alexander Nützel, Nerida G. Wilson, Michael Schrödl

**Affiliations:** 1 Department of Marine Ecosystems Dynamics, Atmosphere and Ocean Research Institute, The University of Tokyo, 5-1-5 Kashiwanoha, Kashiwa, Chiba 277-8564, Japan; 2 SNSB-Bavarian State Collection of Zoology, Münchhausenstr. 21, 81247 München, Germany; 3 Department Biology II, BioZentrum, Ludwig-Maximilians-Universität, Großhadernerstr. 2, 82152 Planegg-Martinsried, Germany; 4 SNSB-Bavarian State Collection of Paleontology and Geology, Geobio Center LMU, Richard-Wagner-Str. 10, 80333 München, Germany; 5 Western Australian Museum, Locked Bag 49, Welshpool DC, Perth, WA 6986, Australia

## Abstract

Euthyneuran gastropods represent one of the most diverse lineages in Mollusca (with over 30,000 species), play significant ecological roles in aquatic and terrestrial environments and affect many aspects of human life. However, our understanding of their evolutionary relationships remains incomplete due to missing data for key phylogenetic lineages. The present study integrates such a neglected, ancient snail family Ringiculidae into a molecular systematics of Euthyneura for the first time, and is supplemented by the first microanatomical data. Surprisingly, both molecular and morphological features present compelling evidence for the common ancestry of ringiculid snails with the highly dissimilar Nudipleura—the most species-rich and well-known taxon of sea slugs (nudibranchs and pleurobranchoids). A new taxon name Ringipleura is proposed here for these long-lost sisters, as one of three major euthyneuran clades with late Palaeozoic origins, along with Acteonacea (Acteonoidea + Rissoelloidea) and Tectipleura (Euopisthobranchia + Panpulmonata). The early Euthyneura are suggested to be at least temporary burrowers with a characteristic ‘bubble’ shell, hypertrophied foot and headshield as exemplified by many extant subtaxa with an infaunal mode of life, while the expansion of the mantle might have triggered the explosive Mesozoic radiation of the clade into diverse ecological niches.

The traditional gastropod subclass Euthyneura is a highly diverse clade of snails and slugs with at least 30,000 living species^1^,^2^. They are ubiquitous in aquatic and terrestrial environments and benefit and harm human life as food, pests, hosts of parasites, and sources of natural products for medical use^3^,^4^,^5^. They also serve as biological models, especially in neuroscience^5^,^6^, and as indicators for environmental conservation and climate change studies^7^,^8^. The traditional classification of Euthyneura, which remains in many contemporary textbooks and biodiversity databases, still recognizes Opisthobranchia (sea slugs and snails) and Pulmonata (lung snails and slugs; see ref. 9 for review). However, molecular phylogenetic analyses have demonstrated the non-monophyly of these taxa^10^,^11^,^12^,^13^ and induced drastic reclassification (reviewed in refs 2,9,14,15).

Recent multi-locus phylogenies recovered three major clades in Euthyneura (*sensu lato*), namely Acteonacea, Nudipleura and Tectipleura (Fig. 1)^15^,^16^. Of these, Acteonacea consists of shelled snails in two small superfamilies, Acteonoidea and Rissoelloidea^15^. Nudipleura is a clade of sea slugs without a shell or with a highly reduced shell; the species-rich, popular and often very colourful Nudibranchia (with the subclades Anthobranchia and Cladobranchia) as well as the less known Pleurobranchoidea belong here^2^,^9^. The last and most diverse clade Tectipleura comprises two reciprocal sister subclades, Euopisthobranchia and Panpulmonata. Euopisthobranchia is most famously represented by sea hares and pteropod sea butterflies but also includes bubble snails in the strict sense (Cephalaspidea *s.s.*)^17^. Panpulmonata encompasses all traditional pulmonates (including common garden snails and slugs) and several, morphologically highly disparate non-pulmonate groups such as sacoglossan sea slugs and ectoparasitic pyramidellid snails^12^,^13^,^15^. Recent phylogenomic studies in principle support these relationships. Although the first, taxon-limited analysis for Euthyneura suggested a paraphyletic Nudipleura^18^, the latest study with a denser sampling has confirmed its monophyletic nature with maximum support^16^. Such substantial changes in phylogenetic hypotheses inevitably entail a fundamental reconsideration of traditional assumptions on the homology of characters and traits of evolution, as well as on the systematics of fossil taxa^9^,^15^.

From a palaeontological point of view, our understanding of euthyneuran evolution based on molecular phylogeny still wants for important elements from key taxa with supposed late Palaeozoic or early Mesozoic origins. Particularly important and yet entirely neglected was the ancient snail family Ringiculidae in its own superfamily Ringiculoidea^19^. Ringiculidae comprises at least several dozens of extant species in such genera as *Ringicula, Ringiculopsis, Ringiculoides* and *Microglyphis*^20^,^21^. They inhabit sand and mud bottoms from the intertidal to abyssal depths worldwide^19^,^22^. The ringiculid anatomy is characterized by a hypertrophied head for burrowing, which is called headshield, and a mid-dorsal siphon (Fig. 1) for directing sand particles upwards whilst burrowing and for exchanging water in the mantle cavity for respiration^22^,^23^,^24^. They feed on interstitial copepod crustaceans and foraminiferans by crushing prey exoskeletons with a specialized portion of the stomach^22^,^25^. The small but often very solid shells of Ringiculidae are recovered in the fossil record from the Middle Jurassic of 161–165 Mya (million years ago)^26^ and they flourished as one of the commonest euthyneuran groups in the Late Cretaceous^27^,^28^. These fossils bear surprising resemblance to living ringiculids and accordingly, most are classified in recent genera, including the oldest, Middle Jurassic *Ringicula buchholzi*^26^.

Historically, ringiculids had been classified in distantly related groups of Gastropoda outside Euthyneura based on conchological characteristics^29^. Succeeding authors classified them as basal members of the ‘opisthobranch’ bubble snails on the grounds that they share the oval shell and headshield (Cephalaspidea *s.l.*; Fig. 1)^19^. Another, entirely different scheme of classification based largely on anatomical characters^30^ recognized the Ringiculidae as closely allied to the similarly bubble-shelled Acteonoidea and Diaphanoidea, which collectively formed Architectibranchia, again outside Euthyneura. This basal position of Ringiculidae away from (Eu)opisthobranchia was confirmed by cladistic analyses of morphological data^25^,^31^. Diaphanoidea was later excluded from Architectibranchia based on multi-locus phylogenies (Fig. 1)^17^,^32^, while none of previous studies had incorporated any molecular data from Ringiculidae, whose position thus remained contentious. An inclusive taxon set representing all major extant lineages is crucial for reconstructing and understanding evolutionary origins and consequences.

Here we present a molecular phylogeny of Euthyneura with the first DNA sequences of Ringiculidae. The new sequences originate from seven ringiculid species that cover all major phenotypes (and thus generic diversity) of the family and were analysed with all presently available data for major euthyneuran clades as well as related outgroup taxa. Our Bayesian and likelihood-based reconstructions clearly reject the original and modern Architectibranchia concepts^25^,^30^,^31^,^32^, but instead indicate an unexpected sister group relationship of ringiculid snails to nudipleuran sea slugs. This once again highlights the enormous evolutionary plasticity of Euthyneura. We furthermore provide microanatomical details derived from 3D reconstruction of serial histology sections to investigate homologies in this sister relationship. Combined with fossil evidence, the new molecular and anatomical data suggest that morphological innovations for burrowing and crawling in soft sediment occurred very early in the evolutionary history of Euthyneura. This represents the plesiomorphic condition, from which various body plans have arisen as a result of succeeding adaptive radiation into diverse aquatic and terrestrial ecosystems.

## Results and Discussion

### Molecular phylogenetic reconstruction

The seven study species of Ringiculidae (Ringiculoidea) formed a robust clade as a well-supported sister group to the Nudipleura in both Bayesian and maximum-likelihood analyses (Fig. 2). This relationship is highly remarkable and counterintuitive; thick-shelled ringiculid snails hardly resemble nudipleuran slugs and their external anatomy also shows many discrepancies (see below). With such dissimilarity, no earlier study suggested their close affinity^9^,^15^. There are two possibilities to explain our tree topology: the phylogenetic reconstruction is erroneous, e.g. suffering from potential long-branch attraction or under-sampling of relevant groups, or ringiculids are genuinely related to nudipleurans but these sister taxa are morphologically different to an unexpected extent.

The quality of a molecular phylogeny depends on careful selection, control and processing of sequence data. We followed best practise procedures^33^: BLAST-checking all novel sequences as well as included data from Genbank, generating and masking alignments with several different settings, and performing multiple phylogenetic analyses under different taxon and data selection regimes. All trees recovered from concatenated genes are highly compatible and most nodes receive maximum Bayesian posterior probability, while bootstrap indices are somewhat lower for basal nodes, as in comparable multi-locus studies^11^,^12^. Also, our sensitivity analyses reveal that the clade of Ringiculidae and Nudipleura is robust against variation of taxon sets, i.e. removing either of the two major nudipleuran subclades, contradicting the assumption of potential long branch attraction [Supplementary Figure S1; note that bootstrap support is even higher (98%) with Pleurobranchoidea alone]. The within-group branches of Ringiculidae are not noticeably longer or shorter than those of Nudipleura or the stem branches leading to the two clades (Fig. 2). Lastly, our topology is largely congruent with the latest phylogenomic phylogeny (without Ringiculidae)^16^ and thus it is not accountable for stochastic, potentially misleading histories of few genes. All these points justify the monophyletic nature of Ringiculidae + Nudipleura, for which we propose a new taxon name Ringipleura.

### Microanatomy and synapomorphies of Ringipleura

Despite the highly different body plans of ringiculids and nudipleurans, we discovered similarity in the nervous system, a suite of morphological characters that are often regarded as crucial for resolving molluscan phylogenetic relationships^30^,^34^. Previous authors postulated that the nervous system of Ringiculidae ‘primitively’ retained a very long and crossing visceral loop with a ganglion on it^20^,^22^,^23^. A crossing visceral nerve loop is plesiomorphic for the entire Gastropoda and represents a configuration called ‘streptoneury’, as the counter-concept to ‘euthyneury’ where the loop is straightened out or shortened^25^,^30^.

However, the concept of a streptoneurous Ringiculidae and hence its phylogenetic position outside Euthyneura^20^,^22^,^23^ were refuted by our 3D reconstruction of semi-thin histological sections. The two crossing nerve cords (Fig. 3b: N1 and N2) are not interconnected posteriorly to each other and therefore do not constitute the visceral loop as previously suggested^20^,^22^,^23^. Instead, the one originating from the left side of the cerebral nerve ring terminates near the anus, and the other, more dorsal one from the right side reaches a ganglion that is associated with the epithelium of a chemosensory organ called the osphradium (Fig. 3b: GO and underlying blue area). This osphradial ganglion had been interpreted as a different kind of ganglion on the visceral loop^20^,^22^, but again the former can be histologically differentiated from the latter in having a more flat form, a deeper stain of neurons and a less distinct separation of the cortex and neuropil in Euthyneura^35^. The dorsal cord can then be regarded as the osphradial nerve, but not a part of a visceral nerve loop (see Haszprunar, 1988: Fig. 3 36). This in turn identifies its swollen anterior root as the supraintestinal ganglion (Fig. 3b,c: G1) and the other cord as the visceral nerve that originates from the true visceral ganglion (G2). The supraintestinal and visceral ganglia are in theory linked to each other, but we were not able to detect such a connective.

Visceral loop ganglia annexed or fused to the cerebropleural ganglia (so that no ganglion remains separate on the loop) have previously been found in many nudipleuran slugs but are otherwise very rare in the gastropod nervous system^34^,^37^. Even more interestingly, the connective between the supraintestinal and visceral ganglia is often lacking in previous descriptions of the nudipleuran nervous system as in our reconstruction for Ringiculidae, while this connective is always thick and easily traced in other euthyneuran groups^35^. Some authors have successfully found this connective in Nudipleura as a very thin nerve running along the much thicker pedal commissure (e.g. ref. 38), potentially explaining the lack of observation by others, as well as in our reconstruction. The approximate course of the hitherto undetected visceral loop in Ringiculidae may be hypothesized as shown in Fig. 3c (yellow line). To conclude, the unique condition of the visceral loop and its ganglia seems to represent supporting evidence of Ringipleura.

A second potential synapomorphy for this clade is the fusion of the head and mantle. Nudipleuran slugs are characterised by their continuous dorsal body wall called the notum, which is formed by the mantle fused to the head and overgrowing the visceral sac (Fig. 2i–k)^34^,^37^. The external anatomy of ringiculid snails superficially shows a close resemblance to acteonoid and euopisthobranch bubble snails, not only by retaining the shell but also in having the headshield for burrowing and crawling in soft sediment (Fig. 2f,g,l,m)^19^. However, we found that the headshield in ringiculids is most likely fused posteriorly to a hypertrophied, everted part of the mantle, and is not solely composed of the head as in acteonoids and euopisthobranchs. The posterior part of the fused ‘headshield’ bears compound defensive glands (Fig. 3a,d: DGL)^22^ that are present in the mantle margin of the latter taxa (orange areas in Fig. 3j,l)^24^,^35^. The separate innervation of anterior and posterior parts of the shield by the cerebropleural ganglia further corroborates the fusion of the head and mantle in Ringiculidae: the anterior part receives four pairs of anteriorly projecting cerebral nerves (Fig. 3a–c: NC) while the posterior part is controlled by more dorsal, presumed pleural or parietal nerves (Fig. 3b,c: NPL; see refs 34,39).

### Internal relationships and divergence times of Ringiculidae

The present molecular phylogeny also provides insights into the evolutionary trends of shell shapes and hence the evaluation of the fossil record of the Ringiculidae. Many species of the family share a distinctive teleoconch morphology with pitted spiral ornaments and a complex aperture that bears multiple columellar folds and a thickened outer lip, as well as a heterostrophic coaxial larval shell^19^,^21^,^27^. Such distinctive and complex characteristics minimize the risk of misidentification of fossil specimens due to convergence. The oldest known ringiculid, *Ringicula buchholzi* from the Callovian (Middle Jurassic) of northeastern Germany, gives a reliable minimum age of the family at 161–165 Mya^26^,^40^. The second oldest *Ringicula blaszyki* was described from the Late Valanginian (Early Cretaceous) of Poland at 134–136 Mya^41^. These Mesozoic species are so similar to the Recent *Ringicula* that the modern representatives of the family can be regarded as ‘living fossils.’ Although inconspicuous in the present era, ringiculids were one of the most flourishing euthyneuran groups in the Cretaceous period^27^,^28^.

In the light of the present phylogeny, however, we assume that the origin of Ringiculidae is actually much older than the ages of the above fossils and that Jurassic or even Triassic ringiculids without the diagnostic apertural characters might have been erroneously placed in other euthyneuran families. The living species of Ringiculidae seem to fall into two major subclades: *Ringiculoides*, and all remaining genera (Fig. 2). *Ringiculoides* is a monotypic genus with the type species *R. kurilensis* occurring on the abyssal plain of the western North Pacific^20^. The shell of *R. kurilensis* is unique among ringiculids in having a proportionally large, oval body whorl with only one columellar fold, a sharp outer lip and a round base without a siphonal canal (Fig. 3e). Interestingly, such a condition of the shell is shared by some of other euthyneuran bubble snails, with particularly similar species in Acteonidae (Fig. 3k)^19^,^21^,^42^. *Ringiculoides* is therefore suggested to retain the plesiomorphic shell morphology of Euthyneura, while the more complex and solid shells of *Ringicula* (including the Jurassic *R. buchholzi*) and its allied genera (Fig. 2c,d) are most plausibly interpreted as an apomorphic condition in the family. Of the derived characters, the terminal thickening of the outer lip seems to have been lost independently in the putatively polyphyletic *Microglyphis* (Figs 3f,I and 4).

Our divergence time estimates based the molecular data and four fossil-based calibration points (see Methods) suggested a late Palaeozoic euthyneuran diversification that leads to the major crown groups including Acteonacea, Tectipleura and Ringipleura (Fig. 4). The divergence between ringiculids and nudipleurans was estimated to date back to 270 Mya of the Permian period [with a 95% highest probability density (HPD) interval of 223–321 Mya]. Sensitivity analyses using only two of the three euthyneuran priors resulted in similar estimates for this split with modes at 252–285 Mya (Supplementary Figure S2). This is approximately the time when several stem groups representing what were formerly called ‘shelled opisthobranchs’ existed, with their first undoubted occurrence in the earliest Triassic of some 250 Mya^40^. These early Mesozoic bubble snails in such extinct families as Acteonellidae, Cylindrobullinidae and Tubiferidae are similar enough in general shell morphology to the living Acteonidae and to the putative plesiomorphic ringiculid *Ringiculoides*. The late Palaeozoic family Acteoninidae with a comparable teleoconch shape might belong to the same stem line of Euthyneura^40^. It can therefore be speculated that some of these early fossils represent stem groups of Ringipleura or Ringiculidae, or even stem nudipleurans retaining external shells.

Regardless of their current taxonomic position in Acteonidae, the Middle Jurassic to Late Cretaceous species of *Tornatellaea* (72–174 Mya) are much less ambiguous members of Ringiculidae with a close resemblance to the Recent *Microglyphis* (see refs 26,27,40,41). The first occurrences of *Tornatellaea* and *Ringicula* agree well with the estimated date of divergence between the two ringiculid subclades at 195 Mya in the Early Jurassic (95% HPD: 141–250 Mya; Fig. 4) with modes in sensitivity analyses at 182–207 Mya (Supplementary Figure S2).

### Bubble-shelled ancestry of Euthyneura and origin of nudipleuran slugs

The topology of the present molecular trees clearly rejects the Architectibranchia concepts, old^30^ or new^32^. A close relationship between Ringiculidae and Acteonoidea^32^ was refuted by the clustering of the latter with Rissoelloidea (Acteonacea; Fig. 2 and see also ref. 16). However, there remains a fundamental uncertainty regarding the position of Ringipleura. The Bayesian reconstruction using MrBayes resulted in an unresolved trichotomy at the base of Euthyneura *sensu lato* (Acteonacea, Ringipleura and Tectipleura; Supplementary Figure S3). The RAxML tree recovered Acteonacea as an unsupported sister to Ringipleura (Fig. 2), while BEAST analyses rendered Acteonacea sister to all other euthyneurans but again with low Bayesian posterior probabilities (Fig. 4). The recent phylogenomic analysis by Zapata *et al.*^16^ resulted in similarly incompatible and poorly supported topologies for the early branching events in the Euthyneura. The three crown groups probably diverged within a relatively short period of time in the late Palaeozoic.

Because of the unresolved basal euthyneuran relationships, it makes little sense yet to reconstruct ancestral character states for ringipleurans in detail. As mentioned above, however, the common ancestor of Euthyneura might have had a relatively thin, oval shell with a large body whorl and a smooth surface with or without pitted spiral ornaments, as seen in the early Mesozoic ‘shelled opisthobranchs’ and Recent bubble snails including *Ringiculoides* and many acteonoideans and cephalaspideans (Fig. 3). Ecologically, such a thin and smooth shell with a large body whorl (hence a large aperture) in shallow marine environments is often associated with an infaunal lifestyle or at least temporary burrowing in the top layer of sediment. Snails with these conchological characteristics are vulnerable to crushing predation and abiotic breakage, which are however less important as a selective agency in soft sediment^43^. On the other hand, a large aperture is most often accompanied by a large foot that enables rapid and efficient burrowing^43^, as does the headshield^22^,^31^. The infaunal mode of life has already been suggested by Brace^44^ for the common ancestor of Euthyneura, from which epifaunal lineages were independently derived after varying intervals of time. We propose that this very plausible hypothesis can be extended to the cause of the parallel shell reduction in Tectipleura. Many infaunal snails, including ringiculids and euopisthobranchs, bear an expanded mantle that partly or entirely covers the shell for further facilitating locomotion (Fig. 3)^31^. We suggest that this relaxed connection between the mantle margin and shell lip, in conjunction with the acquisition of chemical defensive devices^45^, might have triggered the internalization, reduction and loss of the shell for the exploitation of new ecological niches, both within and outside soft sediment, and also on land (see refs 15,46).

**Figure 1:**
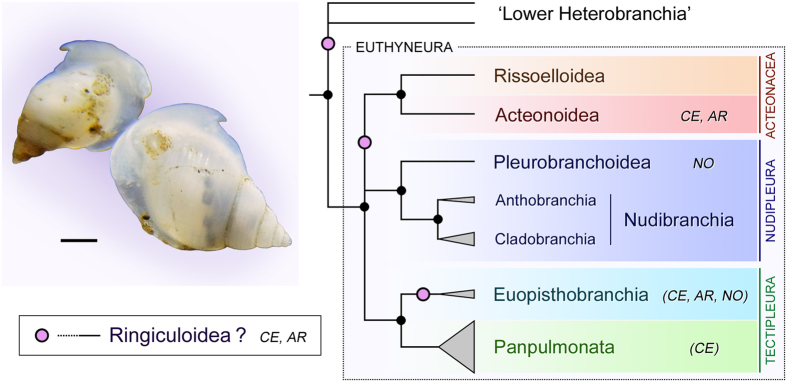
Current consensus phylogeny of Heterobranchia showing relationships among major clades of Euthyneura (after Wägele *et al*.^9^). Black dots indicate strongly supported clades; purple circles denote previously hypothesized positions of Ringiculidae (Ringiculoidea). Vertical height of each triangle represents approximate number of extant species. Acteonoidea, Pleurobranchoidea and Ringiculoidea as well as some subtaxa of Euopisthobranchia and Panpulmonata were traditionally classified in Cephalaspidea (CE), Architectibranchia (AR) or Notaspidea (NO). Left inset shows two live individuals of *Ringicula doliaris* from Kagoshima, Japan (Scale bar: 1 mm).

**Figure 2:**
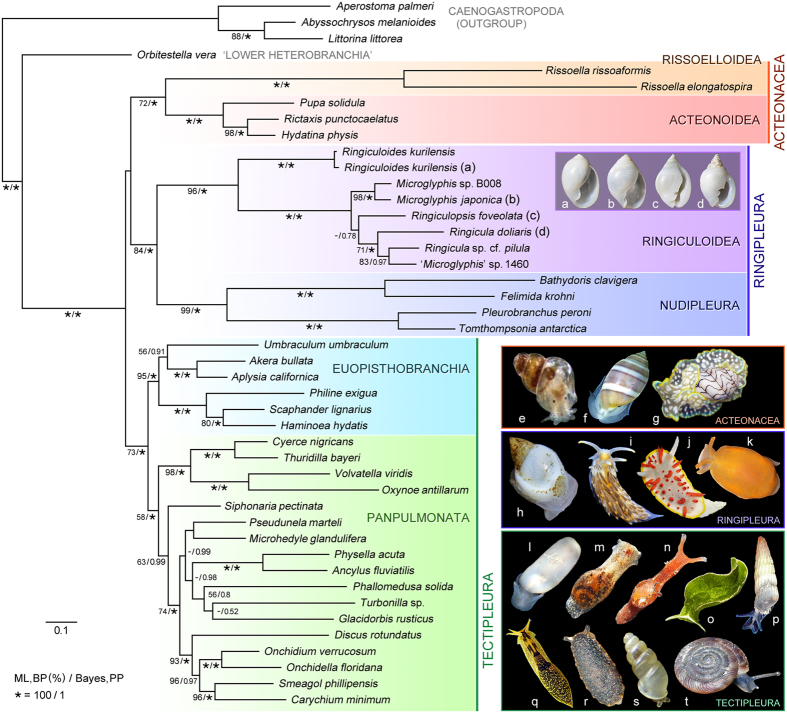
Maximum-likelihood phylogeny of euthyneuran gastropods. Tree reconstruction was performed in RAxML based on combined nucleotide sequences of nuclear 18S and 28S rRNA and mitochondrial 16S rRNA and COI genes (a total of 3,679 sites after exclusion of alignment ambiguous sites). Numerals on branches denote bootstrap values shown as percentages (BP, left) and Bayesian posterior probabilities computed by MrBayes (BPP, right). Significant support in bold (BP ≥ 75%, BPP ≥ 0.99); asterisks denote maximum BP and BPP values (100%, 1.00). **(a–d)** Shells of sequenced Ringiculidae (Ringiculoidea): **(a)** *Ringiculoides kurilensis*, **(b)** *Microglyphis japonica*, **(c)** *Ringiculopsis foveolata* and **(d)** *Ringicula doliaris.* **(e–t)** Live-taken images of representative species of Acteonacea, Ringipleura and Tectipleura: **(e)** *Rissoella opalina*, **(f)** *Acteon tornatilis*, **(g)** *Micromelo undata*, **(h)** *Ringicula doliaris*, **(i)** *Aeolidiella alderi*, **(j)** *Diaphorodoris papillata*, **(k)** *Berthella* sp., **(l)** *Retusa* sp., **(m)** *Haminoea* sp., **(n)** *Aplysia parvula*, **(o)** *Elysia* sp., **(p)** *Turbonilla acutissima*, **(q)** *Acochlidium bayerfehlmanni*, **(r)** *Onchidella celtica*, **(s)** *Carychium pessimum* and **(t)** *Discus rotundatus*.

**Figure 3:**
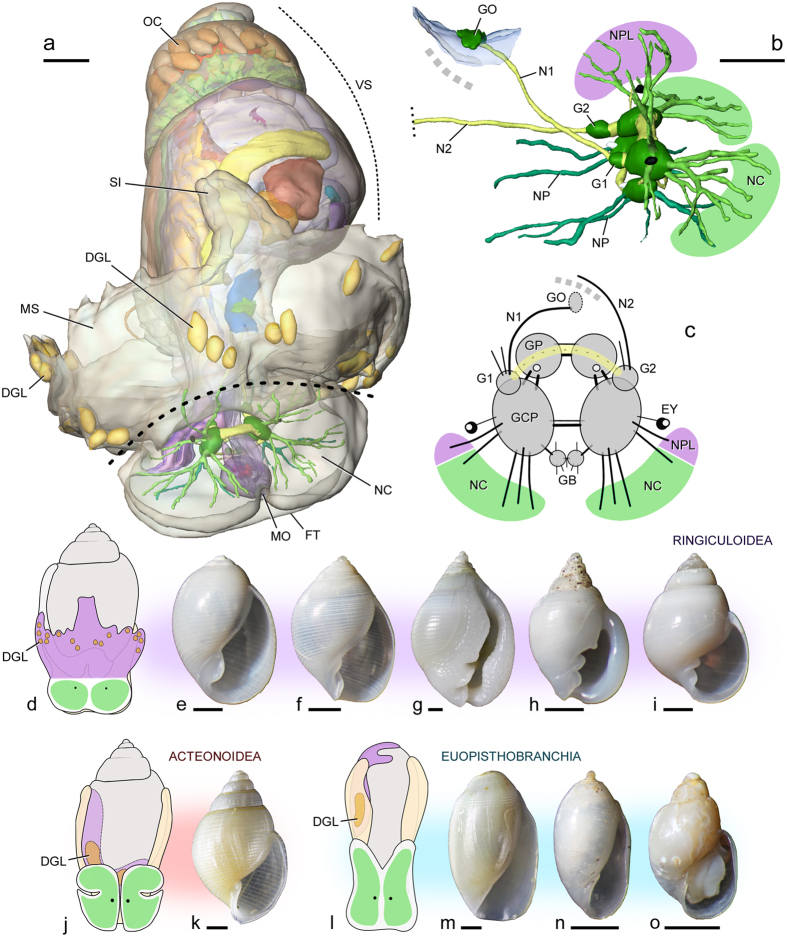
Morphological comparison of euthyneuran bubble snails. **(a–c)** Microanatomy of *Ringicula doliaris* with emphasis on head and nervous system. **(a)** 3D reconstruction of entire animal, anterodorsal view. Broken line indicates separation of headshield into head and mantle parts. **(b)** Central nervous system, oblique right view, highlighting lack of connection between nerves N1 and N2 (grey dotted line). Head (green) and mantle (purple) are innervated by cerebral and pleural (or parietal) nerves, respectively. **(c)** Schematic drawing of central nervous system, orientation as in (**a**). Hypothetical course of visceral loop is shown as yellow line. **(d–i)**. Ringiculidae (Ringiculoidea). **(d)** Schematic drawing of head-hoot, mantle and shell. Green areas denote head with cerebral innervation and purple area represents mantle innervated by pleural or parietal nerves. **(e–i)** Shells of sequenced specimens: **(e)** *Ringiculoides kurilensis*, **(f)** *Microglyphis japonica*, **(g)** *Ringiculopsis foveolata*, **(h)** *Ringicula doliaris* and **(i)** ‘*Microglyphis*’ sp. **(j,k)** Acteonoidea. **(j)** Schematic drawing. Cream area denotes expanded margins of foot or parapodia. **(k)** Shell of a representative species, *Punctacteon teramachii*. **(l–o)** Euopisthobranchia. **(l)** Schematic drawing and shells of **(m)** *Cylichnium ancillarioides*, **(n)** *Acteocina gordonis* and **(o)** *Toledonia* sp. Scale bars: 200 μm for 3D reconstruction; 1 mm for shells. Abbreviations: DGL, defensive gland, seen transparency in (**j,l**); EY, eye; FT, foot; G1, supraintestinal ganglion; G2, visceral ganglion; GB, buccal ganglia; GCP, cerebropleural ganglia; GO, osphradial ganglion; GP, pedal ganglia; MO, mouth; MS, mantle shield; N1, osphradial nerve; N2, visceral nerve; NC, cerebral nerves and innervated area; NP, pedal nerves; NPL, pleural or parietal nerves; OC, oocytes; SI, siphon; VS, visceral sac with internal organs.

The fused head and mantle in Ringipleura (Fig. 3a–d) might have paved the way to the more elaborate and flexible notum of Nudipleura for crawling on a variety of three-dimensional substrates and feeding upon various sessile and mobile invertebrates^37^,^45^. Nudipleura is composed of two reciprocally monophyletic subclades: Nudibranchia and Pleurobranchoidea (Fig. 1). Although the postmetamorphic shell is lacking in all nudibranchs, a very thin, helicoid or plate-like teleoconch is retained under the notum of Pleurobranchidae of the latter subclade^31^. The ontogenetic extension of the mantle over the shell with the eventual inclusion of the latter, described for pleurobranchids^47^, may recapitulate the evolutionary transition from bubble snails to shell-less slugs in Ringipleura. Such fragile shells of Pleurobranchidae are understandably scarce in fossil material. Pacaud *et al.*^48^ mentioned a Palaeocene occurrence (*Berthella* sp.; 62–66 Mya), but this species was neither illustrated nor described in detail. The oldest reliable fossil of the family, hence the whole Nudipleura, dates back only to the late Oligocene (24–26 Mya)^49^. Based on these fossils and on the observation that several basal nudibranchs are restricted to deep or polar waters, Schrödl^50^ suggested the early diversification of Nudipleura was related to the cooling of Antarctica since some 40 Mya. This view was supported and elaborated by Wägele *et al.*^2^, but their hypotheses relied on an assumption that the Nudipleura were phylogenetically close to the externally shelled Umbraculoidea (=Tylodinoidea), which are actually a basal offshoot of Euopisthobranchia (Fig. 2).

**Figure 4:**
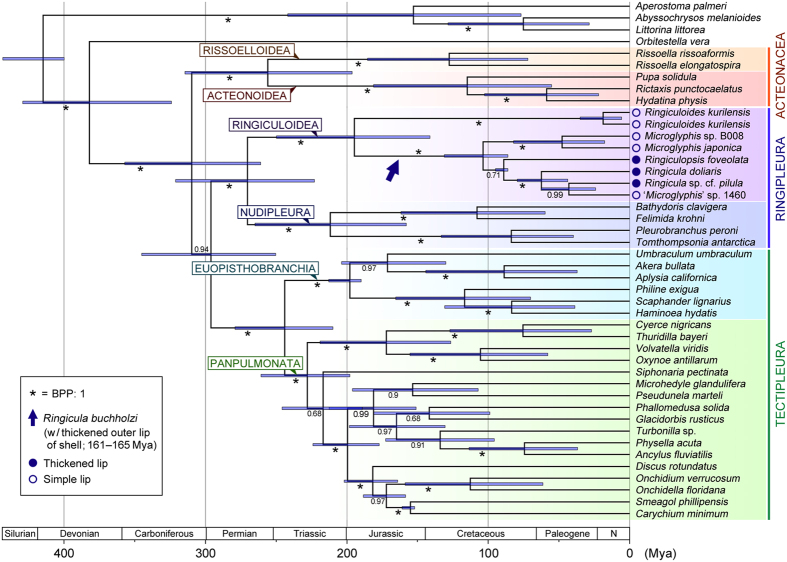
Time-calibrated phylogeny of euthyneuran gastropods. Reconstruction was based on concatenated four-gene sequences (3,679 sites) and four calibration priors on node ages and performed in BEAST. Numerals on branches denote Bayesian posterior probabilities (BPP); asterisks denote maximum value (1.00). Arrow points to age of earliest fossil occurrence of unambiguous Ringiculidae (*Ringicula buchholzi*), which bore a thickened outer lip of shell (plausibly a derived condition within the family). Filled and open circles indicate presence and absence of thickened lip, respectively, in modern ringiculids; note independent losses in putatively polyphyletic *Microglyphis*. Estimated nodal ages (in million years ago, Mya) and 95% credibility intervals (HPD) for first split within clades: Acteonacea, 256 (HPD: 196–315); Ringipleura, 270 (223–321); Tectipleura, 244 (210–279); Rissoelloidea, 128 (72–185); Acteonoidea, 115 (55–181); Ringiculoidea, 195 (141–250); Nudipleura, 212 (158–265); Euopisthobranchia, 198 (190–213); Panpulmonata, 228 (198–261).

More recent time-calibrated phylogenies suggest the origin of the Nudipleura, i.e. their split from Tectipleura or Tectipleura plus Acteonacea, in the Permian or Triassic period^11^,^12^,^16^. Our BEAST analyses resulted in similar dates, despite the inclusion of Ringiculidae as the sister group of Nudipleura. Ringipleura was estimated to have diverged into these subclades at 270 Mya of the Permian (95% HPD: 223–321 Mya; modes in sensitivity analyses at 252–285 Mya) and the first nudipleuran split into Nudibranchia and Pleurobranchoidea at 212 Mya of the Triassic (158–265 Mya and 197–222 Mya, respectively; Fig. 4, Supplementary Figure S2). The internalization, reduction and complete loss of the shell, which are adaptive for actively carnivorous nudipleurans with chemical defence, should thus have occurred during the early to middle Mesozoic to give rise to one of the first slugs in the gastropod evolution–when many other predatory animals originated and diverged in the shallow sea^43^.

## Conclusions

New molecular and anatomical data indicate that ringiculid snails represent an ancient sister clade of nudipleuran sea slugs; a link previously missing to the remaining Euthyneura. The early Euthyneura are suggested to be at least temporary burrowers in soft sediment in the late Palaeozoic, with a characteristic bubble shell with a large body whorl as well as a hypertrophied foot and headshield for the infaunal mode of life. We hypothesize that early euthyneurans relaxed the strict connection of the shell and mantle margin for further facilitating locomotion in soft sediment, thereby releasing the mantle from morphological constraints and allowing the creation of evolutionary novelty, as conceptualized for other animal taxa^51^. This helps to explain the astonishing parallelism found across a number of lineages of euthyneuran slugs and semi-slugs^15^,^46^. Furthermore, the increased flexibility of the body plan might have been a key preadaptive trait behind the explosive Mesozoic radiation of Euthyneura into various ecological niches, including their multiple invasions of the freshwater and terrestrial realms.

## Methods

### Sampling and preparation of specimens

Ringiculid species that cover the generic and conchological diversity of the family were collected from coastal to abyssal waters as shown in Table 1. Most live snails for DNA extraction were boiled in 70–90 °C water for 0.1–1 min and preserved in pure ethanol. The animals of *Ringicula doliaris* for serial sectioning were relaxed in 7.5% magnesium chloride, fixed for 24 hours in a solution of 10% neutral-buffered formalin in sea water, then preserved in 75% ethanol. Voucher material has been deposited at Atmosphere and Ocean Research Institute, The University of Tokyo (AORI), or Bavarian State Collection of Zoology, Germany (ZSM). All shell, radula and cephalic part of the animal were kept undamaged in most specimens for future taxonomic studies.

### DNA extraction, PCR amplification and sequencing

DNA was extracted with DNeasy Blood and Tissue Kit (Qiagen) from the foot tissue of eight ringiculid specimens (Table 1), following the manufacturer’s instructions. Portions of nuclear (18S and 28S rRNA) and mitochondrial (COI and 16S rRNA) genes were amplified using primers shown in Supplementary Table S1; see ref. 52. for amplification conditions and other details. New DNA sequences have been deposited in the DDBJ⁄EMBL⁄GenBank with accession numbers LC150577–LC150593 (Table 1 and Supplementary Table S2). Amplicons were purified by ExoSAP-IT (Affymetrix) following the described protocol. Purified PCR products were sequenced with the amplification and sequencing primers (Supplementary Table S1); sequencing reactions were prepared using a Big Dye Terminator Cycle Sequence Kit 3.1 (Applied Biosystems). The reaction mixtures were analyzed on ABI PRISM 3130xl sequencers after purification with a Big Dye XTerminator Purification Kit (ABI).

### Taxonomic sampling for molecular phylogeny

For phylogenetic analyses of euthyneuran gastropods, we used 44 operational taxonomic units (OTUs) listed in Supplementary Table S2. These include two Rissoelloidea, three Acteonoidea, eight Ringiculidae (Ringiculoidea), four Nudipleura, six Euopisthobranchia and 17 Eupulmonata, as well as four species from the ‘lower Heterobranchia’ and Caenogastropoda for outgroup comparison (see ref. 16). Criteria for our selection of ingroup taxa were (1) the coverage of the phylogenetic diversity of Euthyneura, (2) consistency of evolutionary rates among OTUs, and (3) completeness and accuracy of sequences of all four gene fragments. Many of the lower heterobranch families were not included in our dataset because of the highly accelerated evolutionary rates of their nuclear rRNA and mitochondrial genes and/or the lack of available data. The accuracy of each sequence fragment was checked by BLAST searches and comparison with homologous sequences from related taxa, and species with dubious data were excluded from the succeeding analyses. The final dataset was double-checked by reconstructing single gene trees using the Maximum Likelihood method (see below; Supplementary Figures S4 and S5).

### Sequence alignment and phylogenetic reconstruction

The sequences of the four genes were aligned individually by MAFFT 7.182^53^ with the L-INS-i strategy; the COI sequences were aligned as amino acids. Each aligned dataset was masked to remove alignment ambiguous sites by Gblocks Server 0.91b^54^ with one of three options for a less stringent selection (‘Allow gap positions within the final blocks’).

Phylogenetic trees were reconstructed from a concatenated four-gene dataset using the Bayesian and Maximum-Likelihood (ML) methods in MrBayes 3.1.2^55^ and GUI version of RAxML 7.4.2^56^,^57^, respectively. In the Bayesian analysis, each gene and codon position was allowed to have different parameters, resulting in a total of six unlinked partitions. The model, shape, proportion of invariant sites, state frequency and substitution rate parameters were estimated for each partition (see Supplementary Figure S3). Two parallel runs were made for 10 M generations with a sample frequency of 1,000, using the default value of four Markov chains. The first 5,000 trees for each run were discarded to make sure the four chains reached stationarity by referring to the average standard deviation of split frequencies^55^. The consensus tree and posterior probabilities (BPP) were computed from the remaining 10,000 trees (5,000 trees, two runs). The ML analyses were performed using the same partitions as the Bayesian analysis and following commands: a rapid bootstrap analysis (1,000 replicates) and search for the best-scoring ML tree in a single program run under the default GTR + G model, following the software manual^56^. Bootstrap proportions (BP) of ≥75% and BPP of ≥0.99 were considered significant support.

### Divergence time estimates

The divergence dates between euthyneuran clades and between ringiculid taxa were calculated using the same data set and a relaxed molecular clock model in BEAST 1.5.4^58^. The tree was time-calibrated by setting the ages of the following four nodes: (1) the basal node of the tree, i.e. between Caenogastropoda and Heterobranchia, (2) the first split within Euopisthobranchia, (3) the split between the ellobiid genera *Carychium* and *Smeagol*, and (4) divergence between *Ringiculopsis foveolata* and three other ringiculids. The first calibration point was set at a minimum of 400 million years ago (Mya) with a 95% upper limit of 440 Mya (Gamma distribution, Shape: 1, Offset: 400, Scale: 13.34; see ref. 12), based on the Devonian occurrences of protoconchs characteristic to Caenogastropoda (408–417 Mya) and Heterobranchia (400 Mya)^59^. The second calibration point, the earliest split within the Euopisthobranchia, was set to have a minimum bound of 190 Mya (Scale: 6.33, 95% upper limit: 209 Mya). This interval encompasses the Early Jurassic period, when multiple extant families of euopisthobranch snails first appeared in the fossil record^2^. The third calibration point was constrained at a minimum age of 152 Ma (Scale: 5.07, 95% upper limit: 167.2 Mya) by referring to the earliest fossils of Ellobiidae and phylogenetic relationships within the family^13^,^60^. Lastly, the similar and characteristic shells of the Recent and Cretaceous *Ringiculopsis* (Fig. 3g)^27^,^42^ were considered to justify the long existence of the genus since at least the Santonian age (Offset: 86, Scale: 2.87, 95% upper limit: 94.6 Mya)^28^. Meanwhile, the type genus of the family, *Ringicula*, has even older and more continuous records since the Callovian, Middle Jurassic (161–165 Mya)^26^,^41^. This genus as currently conceived seems to represent a non-monophyletic taxon with plesiomorphic shell features from which some other ringiculid genera had originated, and the Jurassic record therefore could not be used to calibrate the age of a particular node (see Results and Discussion).

The GTR + G model was applied and parameters were unlinked across the six partitions; branch lengths and dates were estimated with an uncorrelated lognormal relaxed-clock model and a Yule prior on the tree. A single run consisted of 100 M generations (with a sample frequency of 1,000) produced 100,000 estimates of divergence dates. The convergence and mixing of the chain were assessed in Tracer 1.5.0 and first 50,000 estimates were discarded as burn-ins. In addition to this main reconstruction with all four calibration points, three separate BEAST analyses without one of the three euthyneuran priors and with 50 M generations were conducted to test the sensitivity of divergence time estimates to possible errors in adopting fossil records (Supplementary Figure S2).

### Microanatomy

Relaxed and formalin-fixed specimens of *Ringicula doliaris* (ZSM Mol 20140460–20140464) were decalcified using Bouin’s fluid, stained in a solution of Safranin in ethanol, dehydrated in an ascending acetone series, and embedded in Epon epoxy resin. Ribbons of serial semithin sections with a thickness of 1.5 to 2 μm were obtained using a Diatome HistoJumbo diamond knife and a Zeiss Microm rotation microtome. Sections were stained using Richardson’s stain and photographed using a ProgRes C3 ccd camera (Jenoptik, Jena, Germany) mounted on a Leica DMB-RBE microscope (Leica Microsystems, Wetzlar, Germany). A 3D reconstruction of the entire body was made for one specimen (ZSM Mol 20140461) from the micrographs (greyscale.*tif*, 1024 × 759 pixels) in Amira 5.2 (Visage Imaging, Berlin, Germany). Presented images are surface renderings or drawings derived from the reconstructed central nervous system. Histology was compared among four sectioned specimens.

## Additional Information

**How to cite this article**: Kano, Y. *et al.* Ringiculid bubble snails recovered as the sister group to sea slugs (Nudipleura). *Sci. Rep.* **6**, 30908; doi: 10.1038/srep30908 (2016).

## Supplementary Material

Supplementary Information

## Figures and Tables

**Table 1:** Ringiculid specimens used in this study.

Species	Locality	Cruise/Station	Coordinates	Depth	Voucher
*Ringicula doliaris*	Nogama Is., Amakusa, Japan		32°35′N, 130°23′E	0–1 m	AORI YK#901
	Ibusuki, Kagoshima, Japan		31°15′N, 130°39′E	0–1 m	ZSM Mol 20140461–0464
*Ringiculia* sp. cf. *pilula*	W of Amami Is., Japan	T/V Nagasaki-maru N319, J-6(4)	28°33′N, 127°02′E	588–621 m	AORI YK#1463
*Ringiculopsis foveolata*	W of Amami Is., Japan	T/V Nagasaki-maru N307, J-6(2)	28°33′N, 127°02′E	606–607 m	AORI YK#1461
*Microglyphis japonica*	E of Kamaishi, Honshu Is., Japan	R/V Tansei-maru KT-12-18, St. 15	39°02′N, 142°24′E	1019–1041 m	AORI YK#2528
*Microglyphis* sp.	SE of Falkland Is., Drake Strait	R/V Polarstern ANTXIX5, PS61/150-1	54°30′S, 56°08′W	286–291 m	ZSM Mol 20140700 (B008)
*‘Microglyphis’* sp.	W of Amami Is., Japan	T/V Nagasaki-maru N319, J-6(5)	28°33′N, 127°02′E	608–631 m	AORI YK#1460
*Ringiculoides kurilensis*	S of Kamchatka, Russia	R/V Hakuho-maru KH-14-2, NBD-1	47°00′N, 160°02′E	5179–5223 m	AORI YK#2531
	SE of Hokkaido Is., Japan	R/V Sonne SO223 (KuramBio), St. 09-09	40°35′N, 150°59′E	5398 m	ZSM Mol 20130355 (B319)

*Ringicula doliaris* from Ibusuki were formalin-fixed and used for microanatomy; others represent pure-ethanol preserved material for molecular phylogeny.
