# Supplementary material for: Ringiculid bubble snails recovered as the sister group to sea slugs (Nudipleura)
Source: Sci Rep. 2016 Aug 8;6:30908. doi: 10.1038/srep30908 (PMC4976385; doi:10.1038/srep30908)
Supplement: Supplementary Information [file srep30908-s1.pdf]

Ringiculid bubble snails recovered as the sister group to sea slugs (Nudipleura)

Yasunori Kano[1], Bastian Brenzinger[2,3], Alexander Nützel[4], Nerida G. Wilson[5] & Michael Schrödl[2,3]

[1]Department of Marine Ecosystems Dynamics, Atmosphere and Ocean Research Institute, The University of Tokyo, 5-1-5 Kashiwanoha, Kashiwa, Chiba 277-8564, Japan. [2]SNSB-Bavarian State Collection of Zoology, Münchhausenstr. 21, 81247 München, Germany. [3]Department Biology II, BioZentrum, Ludwig-Maximilians-Universität, Großhadernerstr. 2, 82152 Planegg-Martinsried, Germany; [4]SNSB-Bavarian State Collection of Paleontology and Geology, Geobio Center LMU, Richard-Wagner-Str. 10, 80333 München, Germany; [5]Western Australian Museum, Locked Bag 49, Welshpool DC, Perth, WA 6986, Australia. Correspondence and requests for materials should be addressed to Y.K. (email: kano@aori.u-tokyo.ac.jp)

Supplementary information:

Table S1. Nucleotide sequences of primers used for amplification and sequencing

Table S2. Gastropod species and sequences used in the phylogenetic reconstruction of euthyneuran clades including Ringiculoidea

Figure S1. RAxML trees based on four-gene dataset without one of two major nudipleuran subclades: (a) Nudibranchia (*Bathydoris* and *Felimida*) and (b) Pleurobranchoidea (*Pleurobranchus* and *Tomthompsonia*).

Figure S2. Time-calibrated BEAST trees without one of four calibration priors: (a) first split within Euopisthobranchia and (b) splits between *Carychium* and *Smeagol* and (c) between *Ringiculopsis* and three other ringiculids.

Figure S3. MrBayes tree based on four-gene dataset.

Figure S4. RAxML trees inferred from independent gene fragments: (a) 18S rDNA, (b) 28S rDNA, (c) 16S rDNA and (d) COI.

Figure S5. RAxML trees inferred from combined nuclear or mitochondrial gene dataset: (a) nuclear 18S and 28S; (b) mitochondrial 16S and COI.

Kano et al. Table S1. Nucleotide sequences of primers used for amplification and sequencing.

Locus	Primer	Sequence	Direction	Position[*1]	Reference
18S	18A1	CCTACCTGGTTGATCCTGCCAG	Forward	–21 to 0	Steiner and Dreyer (2003)
	188f	GGATCTATTGGAGGGCAAGT	Forward	554–573	Nakamura et al. (2007)
	NS2[*2]	GGCTGCTGGCACCAGACTTGC	Reverse	568–588	White et al. (1990)
	NS5	AACTTAAAGGAATTGACGGAAG	Forward	1168–1189	White et al. (1990)
	189r	TCGGAATTAACCAGACAAATC	Reverse	1335–1355	Nakamura et al. (2007)
	1800r	ATGATCCTTCCGCAGGTTCACC	Reverse	1957–1978	Steiner and Dreyer (2003)
28S	LSU5	TAGGTCGACCCGCTGAAYTTAAGCA	Forward	–33 to –9	Littlewood et al. (2000)
	900F	CCGTCTTGAAACACGGACCAAG	Forward	636–657	Lockyer et al. (2003)
	ECD2S	CTTGGTCCGTGTTTCAAGACGG	Reverse	636–657	Williams and Ozawa (2006)
	LSU1600R	AGCGCCATCCATTTTCAGG	Reverse	1024–1042	Williams et al. (2003)
COI–16S	LCO1490	GGTCAACAAATCATAAAGATATTGG	Forward	–24 to 0	Folmer et al. (1994)
	COIf[*2]	CCTGCAGGAGGAGGAGAYCC	Forward	617–636	This study
	COIf-A[*2]	CCTGCTGGTGGAGGTGAYCC	Forward	617–636	This study
	COIf-B[*2]	CCTGCTGGTGGTGGAGAYCC	Forward	617–636	This study
	COIf-G[*2]	CCAGCTGGWGGGGGTGATCC	Forward	617–636	This study
	HCO2198	TAAACTTCAGGGTGACCAAAAAATCA	Reverse	659–684	Folmer et al. (1994)
	Opis COI-Fm	ACTTTTTTTCCTCAACATTTYTT	Forward	1220–1242	Modified from Grande et al. (2002)
	COIa-NER	CATTTAGTGTAGCAATCAGGRTARTC	Reverse	1274–1299	Kano and Kase (2004)
	16Sar-L	CGCCTGTTTATCAAAAACAT	Forward	2017–2036	Palumbi et al. (1991)
	Opis1-R	ATTAYGCTACCTTAGCACRGTCA	Reverse	2098–2120	Grande et al. (2002)
	16Sbr-H	CCGGTCTGAACTCAGATCAYGT	Reverse	2458–2479	Modified from Palumbi et al. (1991)

[*1]Position of primers on amplified sequences of *'Microglyphis'* sp. (AORI YK#1460; 18S: LC150579) or *Ringicula doliaris* (#901; 28S: LC150580; COI–16S: LC150582)

[*2]Sequencing primers

References

Folmer, O., Black, M., Hoeh, W., Luts, R., Vrijenhoek, R., 1994. DNA primers for amplification of mitochondrial cytochrome c oxidase subunit I from diverse metazoan invertebrates. Mol. Mar. Biol. Biotechnol. 3, 294–299.

Grande, C., Templado, J., Lucas Cervera, J., Zardoya, R. 2004. The complete mitochondrial genome of the nudibranch Roboastra europaea (Mollusca: Gastropoda) supports the monophyly of opisthobranchs. Mol. Biol. Evol. 21, 303–313.

Kano, Y., Kase, T. 2004. Genetic exchange between anchialine cave populations by means of larval dispersal: the case of a new gastropod species *Neritilia cavernicola*. Zool. Scr. 33, 423–437.

Littlewood, D.T.J., Curini-Galletti, M., Herniou, A., 2000. The interrelationships of Proseriata (Platyhelminthes: Seriata) tested with molecules and morphology. Mol. Phylogenet. Evol. 16, 449–466.

Lockyer, A.E., Olson, P.D., Østergaard, P., Rollinson, D., Johnston, D.A., Attwood, S.W., Southgate, V.R., Horak, P., Snyder, S.D., Le, T.H., Agatsuma, T., McManus, D.P., Carmichael, A.C., Naem, S., Littlewood, D.T.J., 2003. The phylogeny of the Schistosomatidae based on three genes with emphasis on the interrelationships of *Schistosoma* Weinland, 1858. Parasitology 126, 203–224.

Nakamura, K., Kano, Y., Suzuki, N., Namatame, T., Kosaku, A. 2007. 18S rRNA phylogeny of sea spiders with emphasis on the position of Rhynchothoracidae. Mar. Biol., 153, 213–223.

Palumbi, S., Martin, A., Romano, S., McMillan, W.O., Stice, L., Grabowski, G., 1991. The Simple Fool’s Guide to PCR, Version 2.0. Department of Zoology and Kewalo Marine Laboratory, University of Hawaii, Honolulu.

Steiner, G., Dreyer, H., 2003. Molecular phylogeny of Scaphopoda (Mollusca) inferred from 18S rRNA sequences—support for a Scaphopoda–Cephalopoda clade. Zool. Scr. 32, 343–356.

White, T.J., Bruns, T., Lee, S., Taylor, J.W., 1990. Amplification and direct sequencing of fungal ribosomal RNA genes for phylogenetics, in: Innis, M.A., Gelfand, D.H., Sninsky, J.J., White, T.J. (Eds.), PCR Protocols: a Guide to Methods and Applications. Academic Press, New York, pp. 315–322.

Williams, S.T., Ozawa, T., 2006. Molecular phylogeny suggests polyphyly of both the turban shells (family Turbinidae) and the superfamily Trochoidea (Mollusca: Vetigastropoda). Mol. Phylogenet. Evol. 39, 33–51.

Williams, S.T., Reid, D.G., Littlewood, D.T.J., 2003. A molecular phylogeny of the Littorininae (Gastropoda: Littorinidae): unequal evolutionary rates, morphological parallelism, and biogeography of the southern ocean. Mol. Phylogenet. Evol. 28, 60–86.

Kano et al. Table S2. Gastropod species and sequences used in the phylogenetic reconstruction of euthyneuran clades including Ringiculoidea. DDBJ/EMBL/GenBank accession number and length of sequences are shown along with the voucher of sequenced specimens.

Clade	Family	Species	18S	length	28S	length	16S	length	COI	length	Voucher
Caenogastropoda	Cyclophoridae	*Aperostoma palmeri*	DQ093435	1791	DQ279983	1951	DQ093479	519	DQ093523	657	
	Abyssochrysidae	*Abyssochrysos melanioides*	AB930376	1765	AB930325	2127	AB930403	475	AB930459	630	AORI YK#1482
	Littorinidae	*Littorina littorea*	X91970	1831	AJ488672	1443	DQ093481	519	AJ622946	1199	
Orbitestelloidea	Orbitestellidae	*Orbitestella vera*	FJ917207	1793	FJ917239	1032	FJ917250	479	FJ917268	580	EED-Phy-518
Rissoelloidea	Rissoellidae	*Rissoella rissoaformis*	FJ917214	1418	FJ917226	3681	FJ917252	403	FJ917271	577	EED-Phy-502
		Rissoella elongatospira	FJ917203	2175	FJ917232	1106	–		FJ917270	577	EED-Phy-501
Acteonoidea	Acteonidae	*Pupa solidula*	AY427516	1842	AY427481	1065	EF489319*	429	DQ238006	593	*EED-Phy-35
		Rictaxis punctocaelatus	EF489346	1387	FJ917243	2536	EF489318	365	EF489393	624	EED-Phy-454
					/EF489370	1071					
	Hydatinidae	*Hydatina physis*	AY427515	1804	AY427480	1087	EF489320*	420	DQ991932	14153	*EED-Phy-37
**Ringiculoidea**	**Ringiculidae**	***Ringicula doliaris***	**LC150577**	**1304**	**LC150580**	**1014**	**LC150582**	**2457**	**LC150582**	**2457**	**AORI YK#901**
		Ringiculia* sp. cf. *pilula	**–**		**–**		**LC150583**	**2462**	**LC150583**	**2462**	**AORI YK#1463**
		Ringiculopsis foveolata	**LC150578**	**1315**	**LC150581**	**929**	**LC150584**	**2520**	**LC150584**	**2520**	**AORI YK#1461**
		Microglyphis japonica	**–**		**–**		**LC150587**	**425**	**LC150586**	**658**	**AORI YK#2528**
		***Microglyphis* sp.**	**–**		**–**		**LC150589**	**441**	**LC150588**	**658**	**ZSM Mol 20140700**
		***'Microglyphis'* sp.**	**LC150579**	**1956**	**–**		**LC150585**	**2470**	**LC150585**	**2470**	**AORI YK#1460**
		Ringiculoides kurilensis	**–**		**–**		**LC150591**	**438**	**LC150590**	**1273**	**AORI YK#2531**
			**–**		**–**		**LC150593**	**440**	**LC150592**	**658**	**ZSM Mol 20130355**
Nudipleura	Bathydorididae	*Bathydoris clavigera*	AY165754	2064	AY427444	1383	AF249222	445	AF249808	599	
	Chromodorididae	*Felimida krohni*	AJ224774	1887	AY427445	1064	AY345036	2647	AY345036	2647	
	Pleurobranchidae	*Pleurobranchus peroni*	AY427494	2022	AY427455	1120	EF489331*	443	DQ237993	593	*EED-Phy-436

		Tomthompsonia antarctica	AY427492	2105	AY427452	1156	EF489330*	440	DQ237992	593	*EED-Phy-435
Umbraculoidea	Umbraculidae	*Umbraculum umbraculum*	AY165753	1843	FJ917246*	2375	EF489322*	439	AY345023	1360	*EED-Phy-51
		(*U. mediterraneum*)			/AY427457	1069					
Anaspidea	Akeridae	*Akera bullata*	AY427502	1789	AY427466	1047	AF156127	418	AF156143	658	
	Aplysiidae	*Aplysia californica*	AY039804	1802	AY026366	3951	AF192295	422	AF077759	658	
Cephalaspidea	Philinidae	*Philine exigua*	HQ168425	1798	HQ168438	1036	HQ168412	420	HQ168450	624	ZSM Mol 20080752
	Scaphandridae	*Scaphander lignarius*	EF489348*	1788	KC351544	1488	EF489324*	441	DQ974663	698	*EED-Phy-442
	Haminoeidae	*Haminoea hydatis*	AY427504	1847	AY427468	1037	EF489323*	425	DQ238004	593	*EED-Phy-421
Sacoglossa	Oxynoidae	*Oxynoe antillarum*	FJ917441	1998	FJ917247*	2411	FJ917425	487	FJ917483	593	*EED-Phy-723
					/FJ917466	1085					
	Volvatellidae	*Volvatella viridis*	HQ168426	1930	HQ168439	1074	HQ168413	435	HQ168451	657	AORI YK#890
	Caliphyllidae	*Cyerce nigricans*	AY427500	1847	AY427463	1063	EU140843	441	DQ237995	593	
	Plakobranchidae	*Thuridilla bayeri*	AF249220	1847	AY427461	1058	DQ480206	434	DQ471271	618	
Siphonarioidea	Siphonariidae	*Siphonaria pectinata*	HQ659934	1785	DQ256744	2931	AY377627	438	AF120638	669	
Glacidorboidea	Glacidorbidae	*Glacidorbis rusticus*	FJ917211	1879	FJ917227	3493	FJ917264	454	FJ917284	577	EED-Phy-881
Pyramidelloidea	Pyramidellidae	*Turbonilla* sp.	EF489351	1838	EF489376	1004	EF489332	473	EF489396	621	EED-Phy-526
Amphiboloidea	Phallomedusidae	*Phallomedusa solida*	DQ093440	1816	DQ279991	2081	DQ093484	442	DQ093528	654	
Hygrophila	Physidae	*Physella acuta*	AY282600	1708	EF489368*	1011	AY651241	494	JQ390525	14490	*SMF 325459
	Planorbinae	*Ancylus fluviatilis*	AY282593	1707	EF489365*	1042	EF489312*	427	AY282582	959	*SMF 325462
Acochlidia	Parhedylidae	*Microhedyle glandulifera*	HQ168437	1796	HQ168449	1046	HQ168424	441	HQ168461	655	ZSM Mol 20081019
	Pseudunelidae	*Pseudunela marteli*	HQ168431	1795	HQ168444	1032	HQ168418	438	HQ168456	655	ZSM Mol 20080393
Eupulmonata	Discidae	*Discus rotundatus*	FJ917212	1854	FJ917240	1052	FJ917265	425	FJ917285	577	EED-Phy-607
	Onchidiidae	*Onchidium verruculatum*	Y427522	1791	AY427487	1050	EF489316*	486	EF489391*	626	*EED-Phy-38
		Onchidella floridana	AY427521	1790	AY427486	1049	EF489317*	492	EF489392*	619	*EED-Phy-462
	Ellobiidae	*Smeagol phillipensis*	FJ917210	1840	FJ917229	3399	FJ917263	425	FJ917283	577	EED-Phy-878
		Carychium minimum	EF489341	1739	EF489361	1061	EF489308	465	EF489386	663	

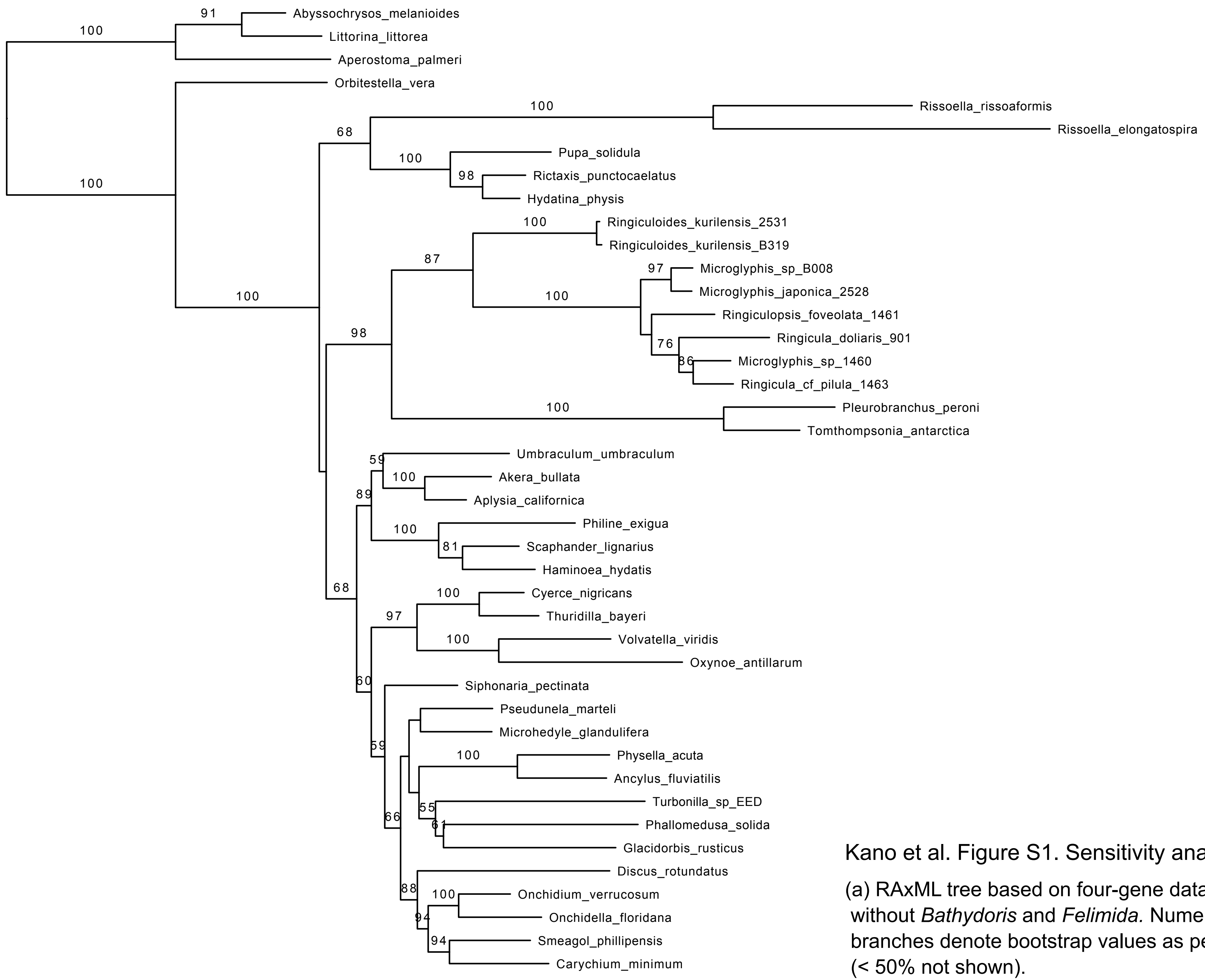

Kano et al. Figure S1. Sensitivity analysis.

(a) RAxML tree based on four-gene dataset without *Bathydoris* and *Felimida.* Numerals on branches denote bootstrap values as percentages (< 50% not shown).

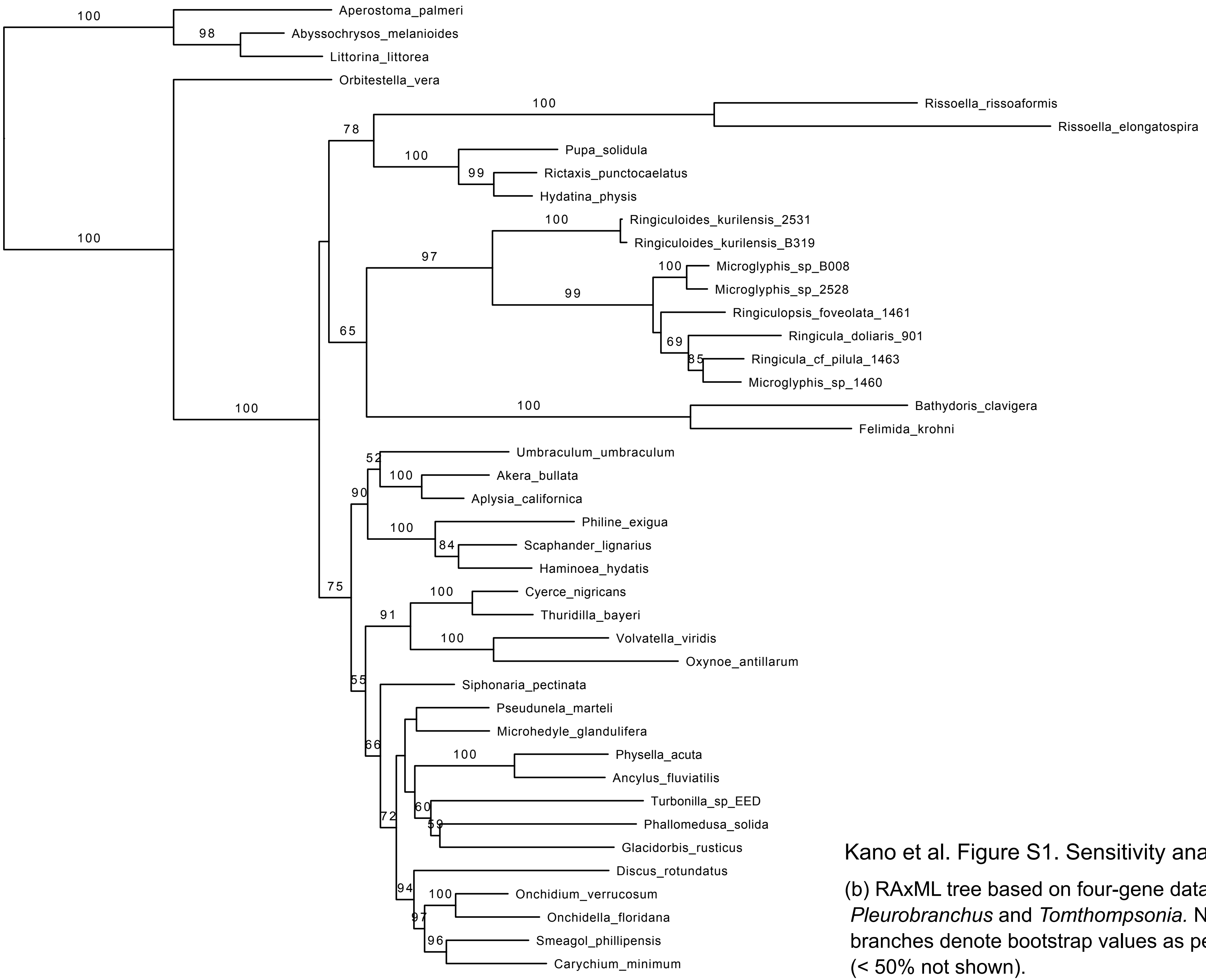

Kano et al. Figure S1. Sensitivity analysis.

(b) RAxML tree based on four-gene dataset without *Pleurobranchus* and *Tomthompsonia.* Numerals on branches denote bootstrap values as percentages (< 50% not shown).

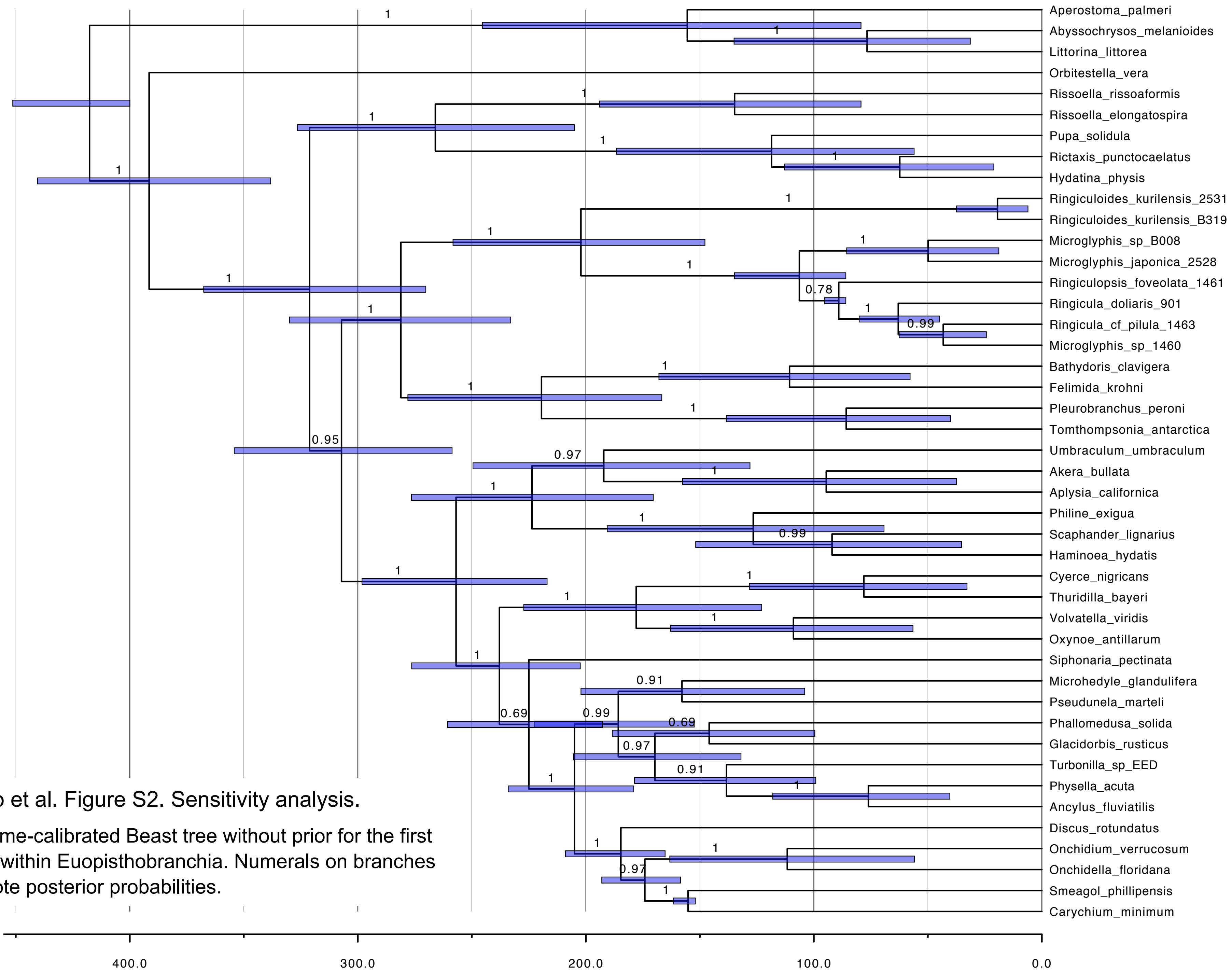

Kano et al. Figure S2. Sensitivity analysis.

(a) Time-calibrated Beast tree without prior for the first split within Euopisthobranchia. Numerals on branches denote posterior probabilities.

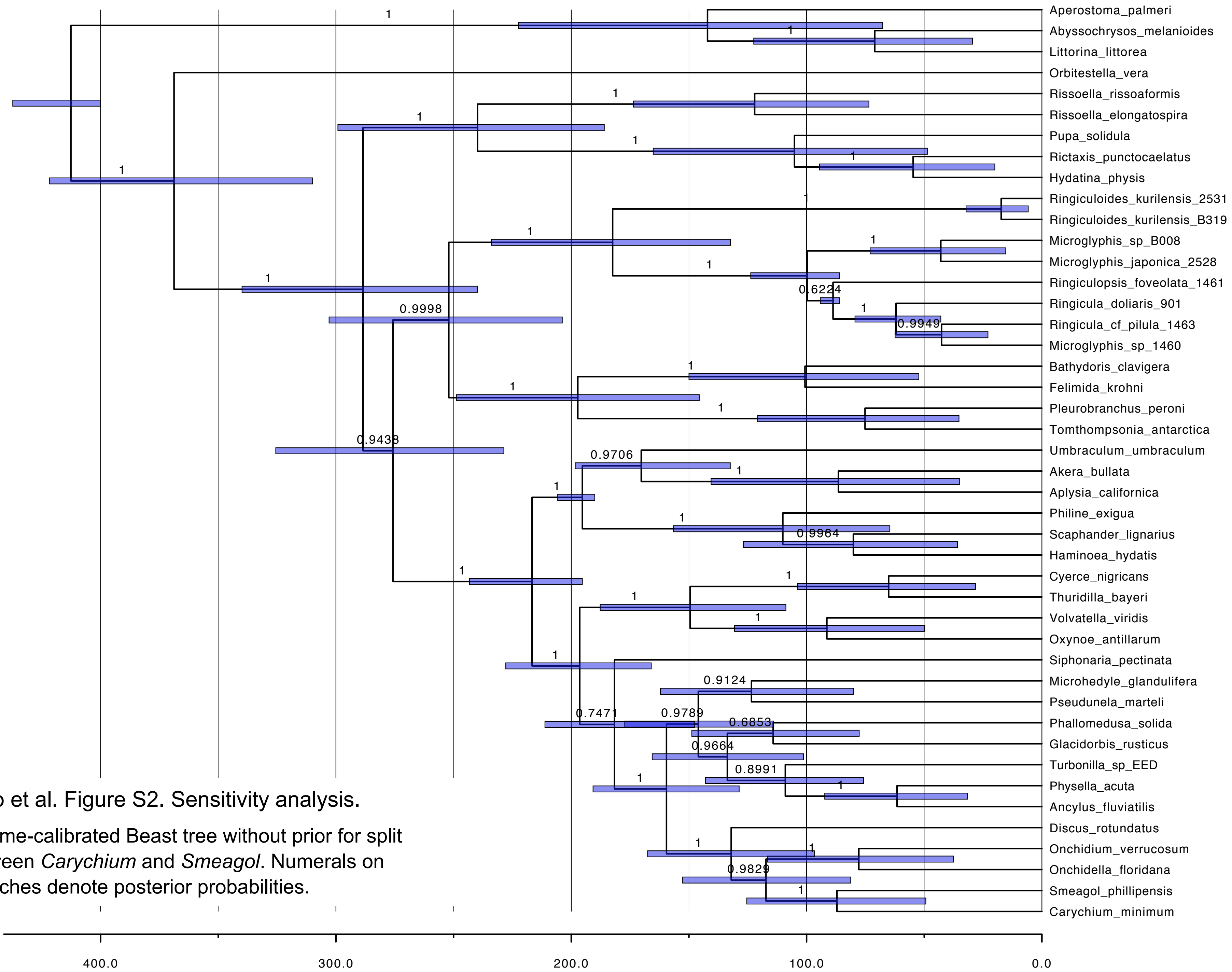

Kano et al. Figure S2. Sensitivity analysis.

(b) Time-calibrated Beast tree without prior for split between *Carychium* and *Smeagol*. Numerals on branches denote posterior probabilities.

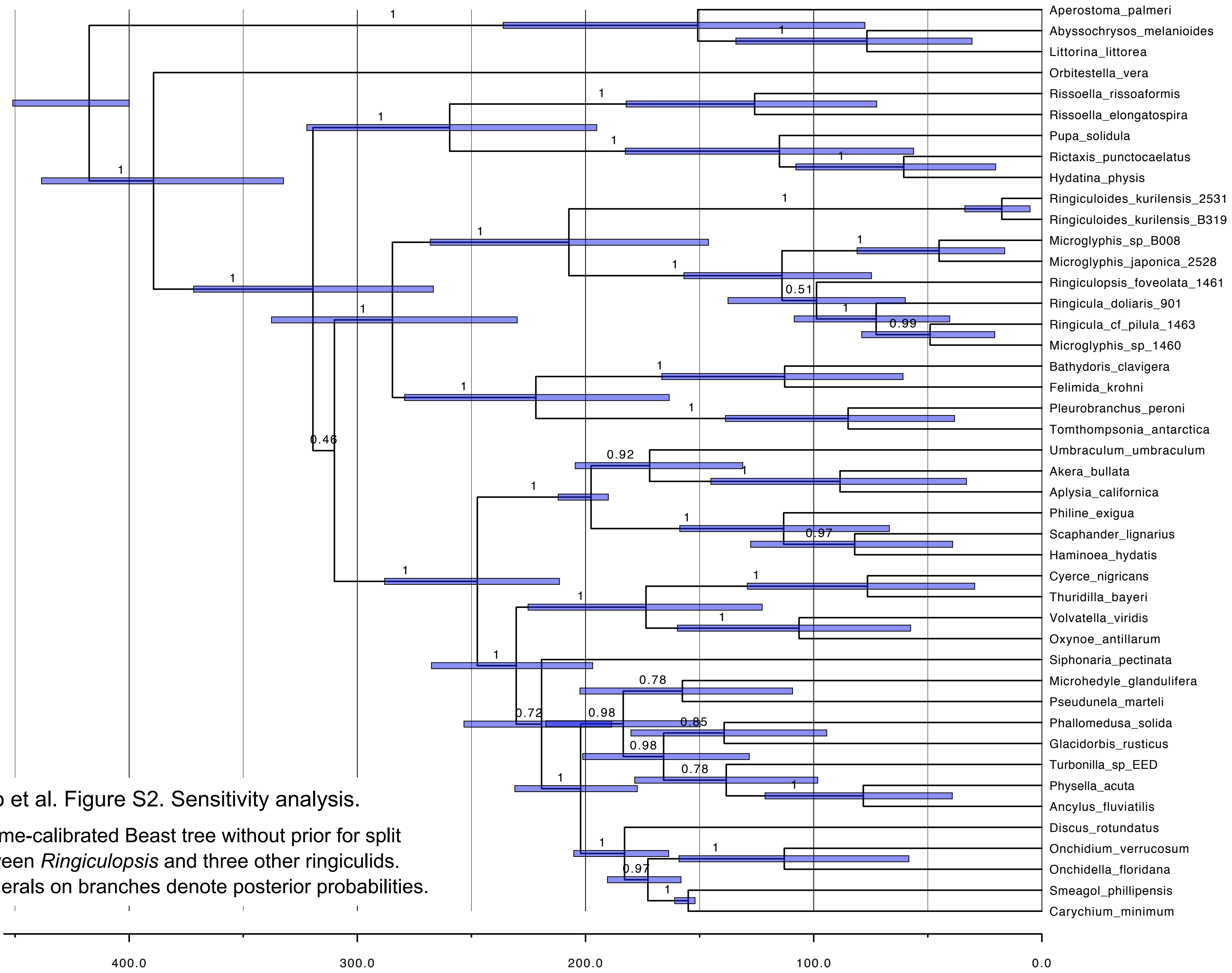

Kano et al. Figure S2. Sensitivity analysis.

(c) Time-calibrated Beast tree without prior for split between *Ringiculopsis* and three other ringiculids. Numerals on branches denote posterior probabilities.

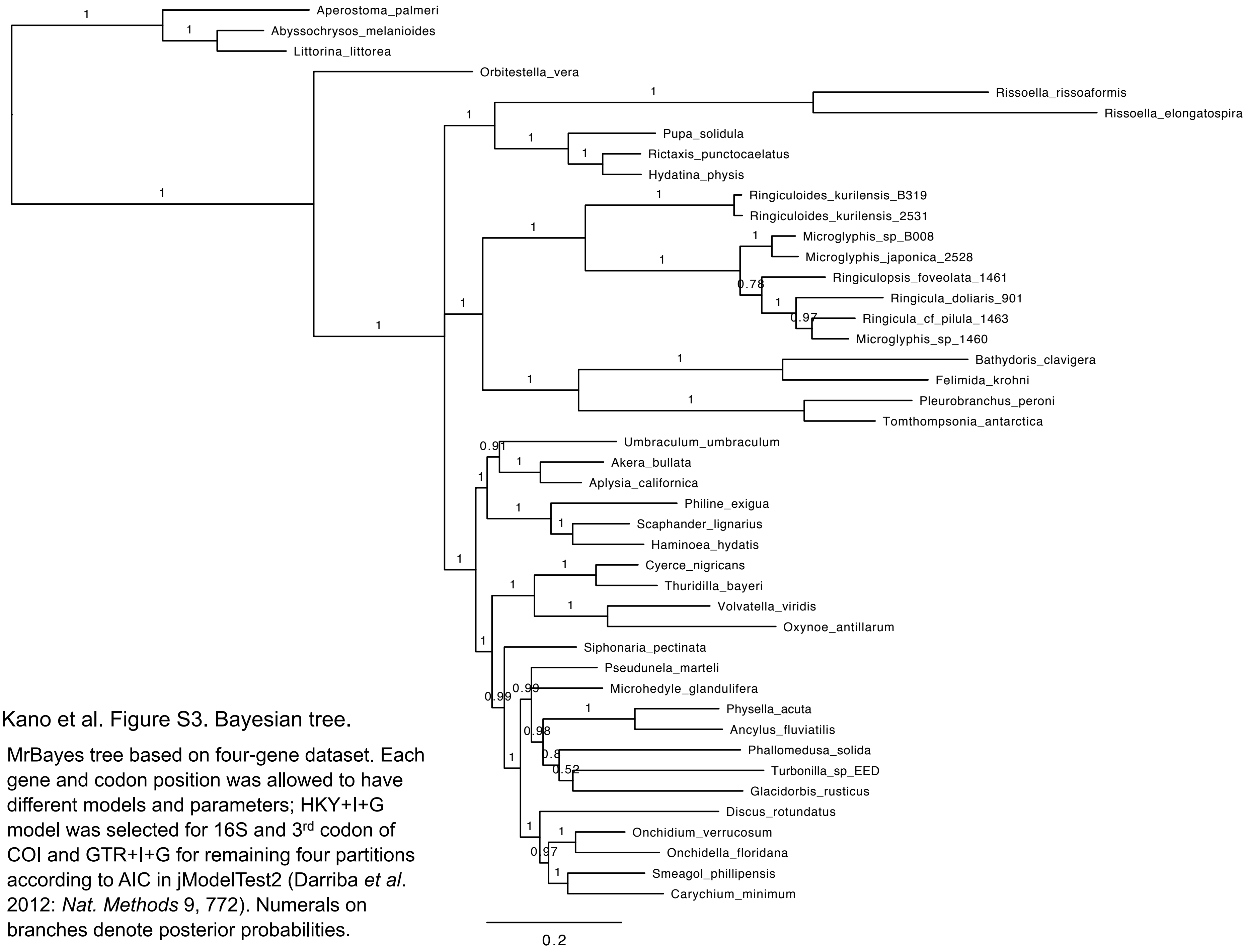

Kano et al. Figure S3. Bayesian tree.

MrBayes tree based on four-gene dataset. Each gene and codon position was allowed to have different models and parameters; HKY+I+G model was selected for 16S and 3rd codon of COI and GTR+I+G for remaining four partitions according to AIC in jModelTest2 (Darriba *et al*. 2012: *Nat. Methods* 9, 772). Numerals on branches denote posterior probabilities.

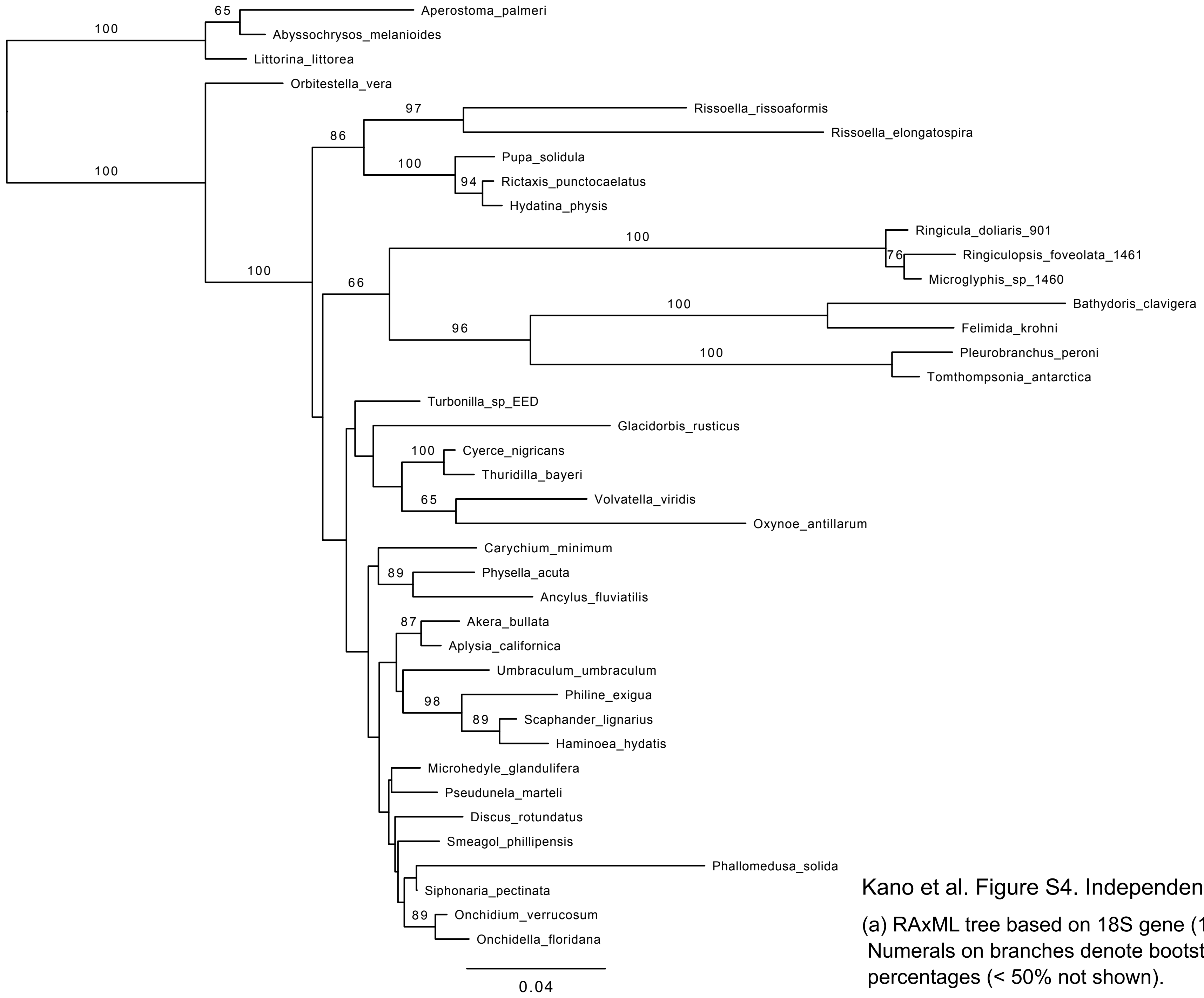

Kano et al. Figure S4. Independent gene trees.

(a) RAxML tree based on 18S gene (1,707 sites). Numerals on branches denote bootstrap values as percentages (< 50% not shown).

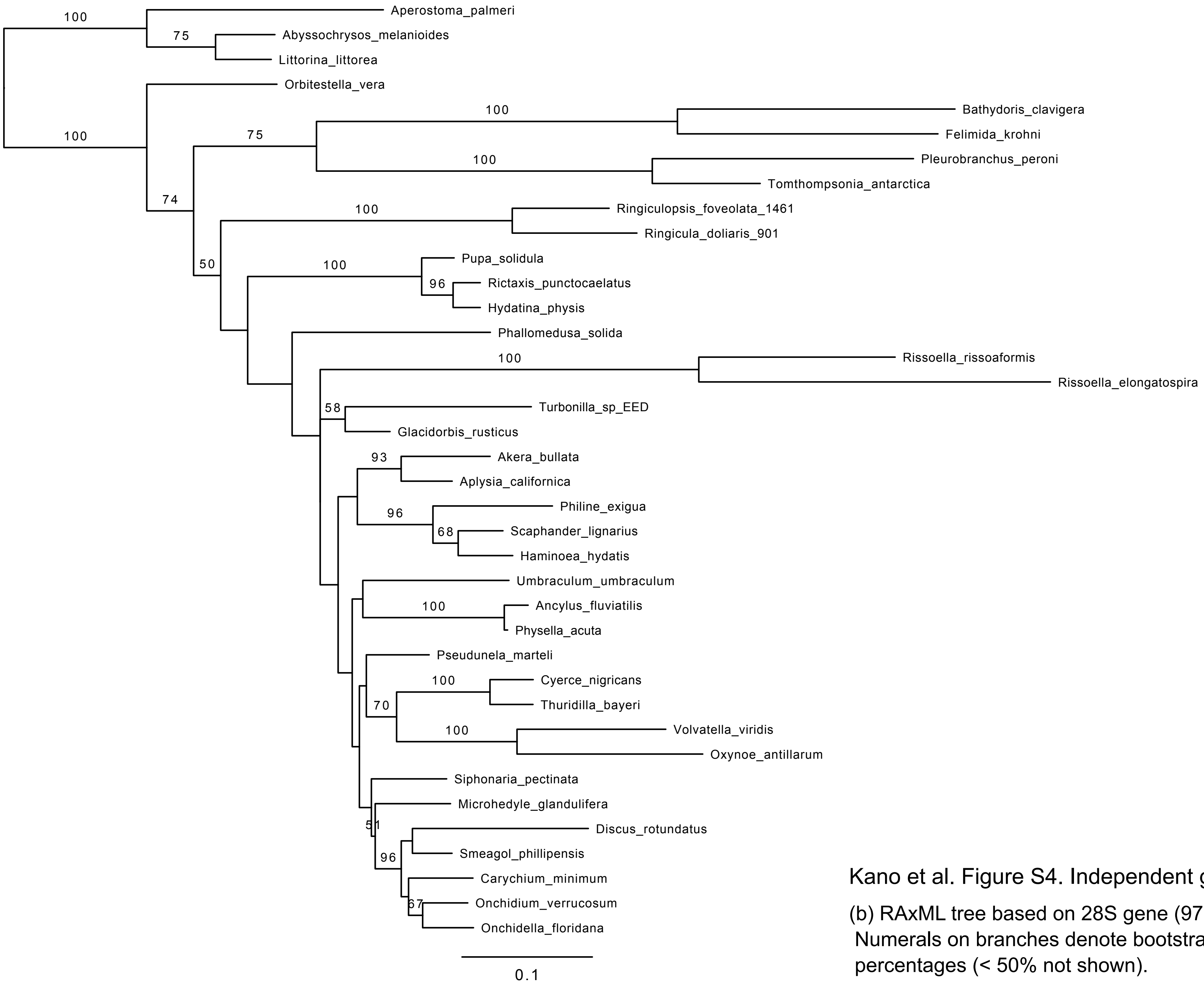

Kano et al. Figure S4. Independent gene trees.

(b) RAxML tree based on 28S gene (973 sites). Numerals on branches denote bootstrap values as percentages (< 50% not shown).

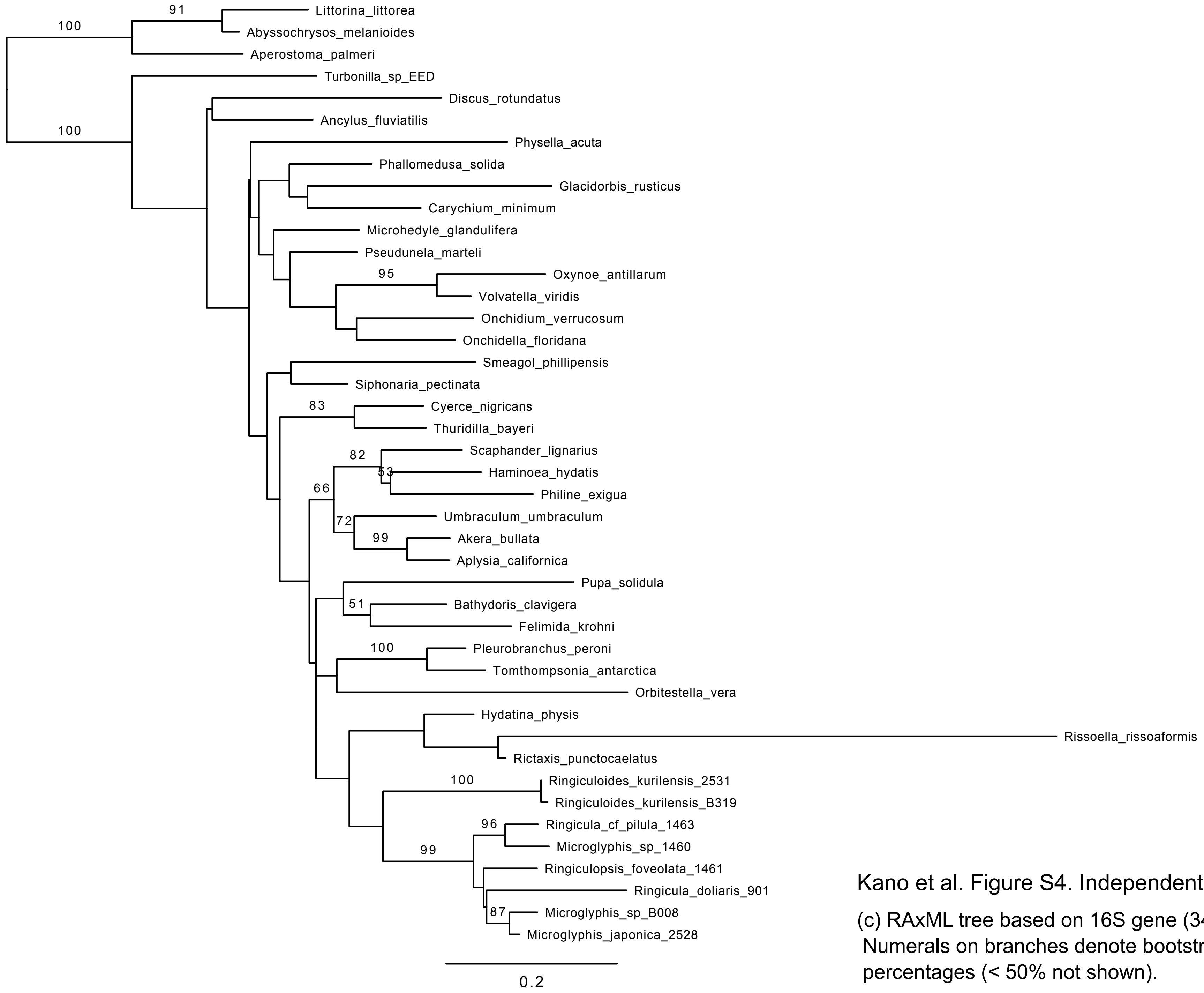

Kano et al. Figure S4. Independent gene trees.

(c) RAxML tree based on 16S gene (342 sites). Numerals on branches denote bootstrap values as percentages (< 50% not shown).

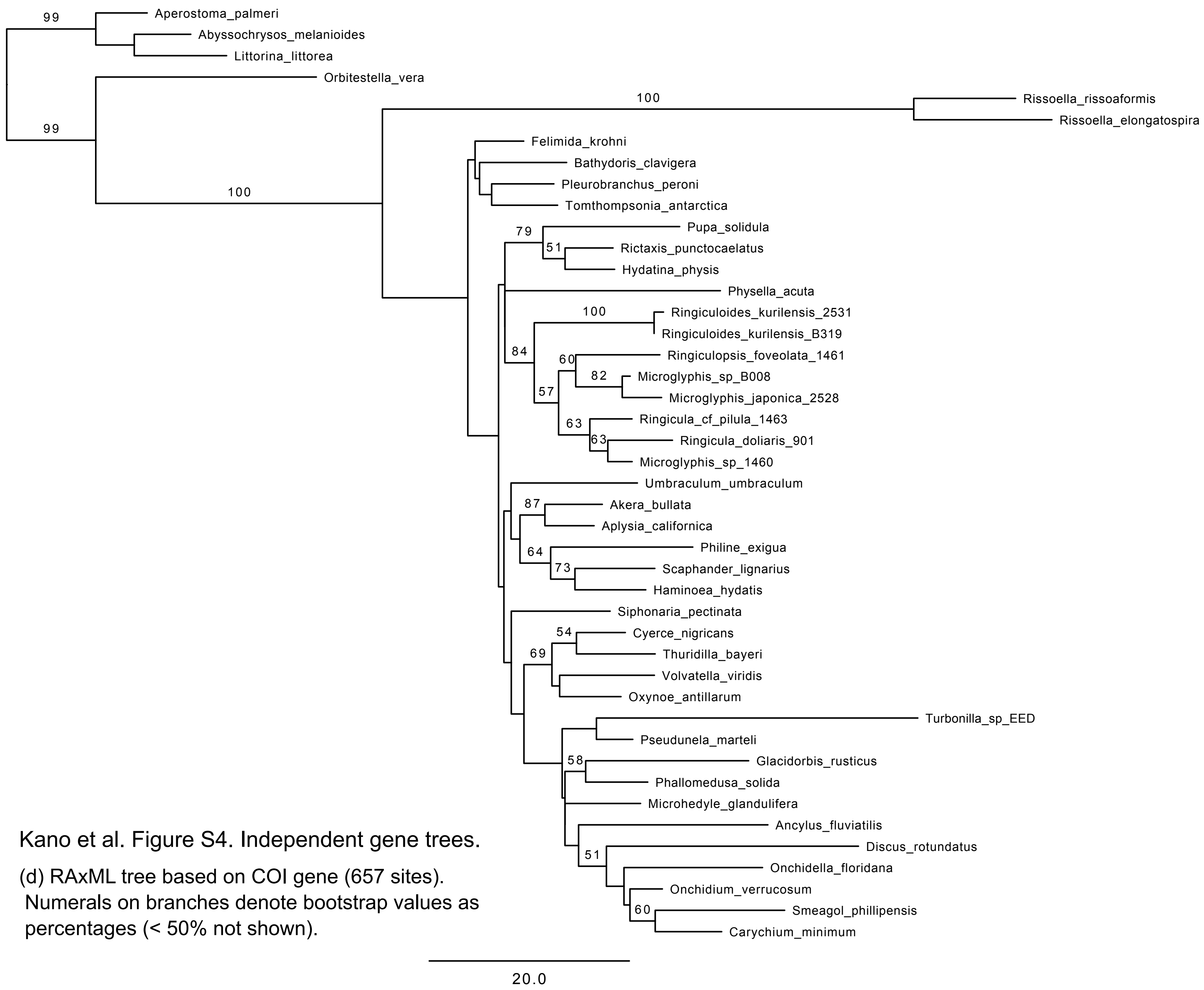

Kano et al. Figure S4. Independent gene trees.

(d) RAxML tree based on COI gene (657 sites). Numerals on branches denote bootstrap values as percentages (< 50% not shown).

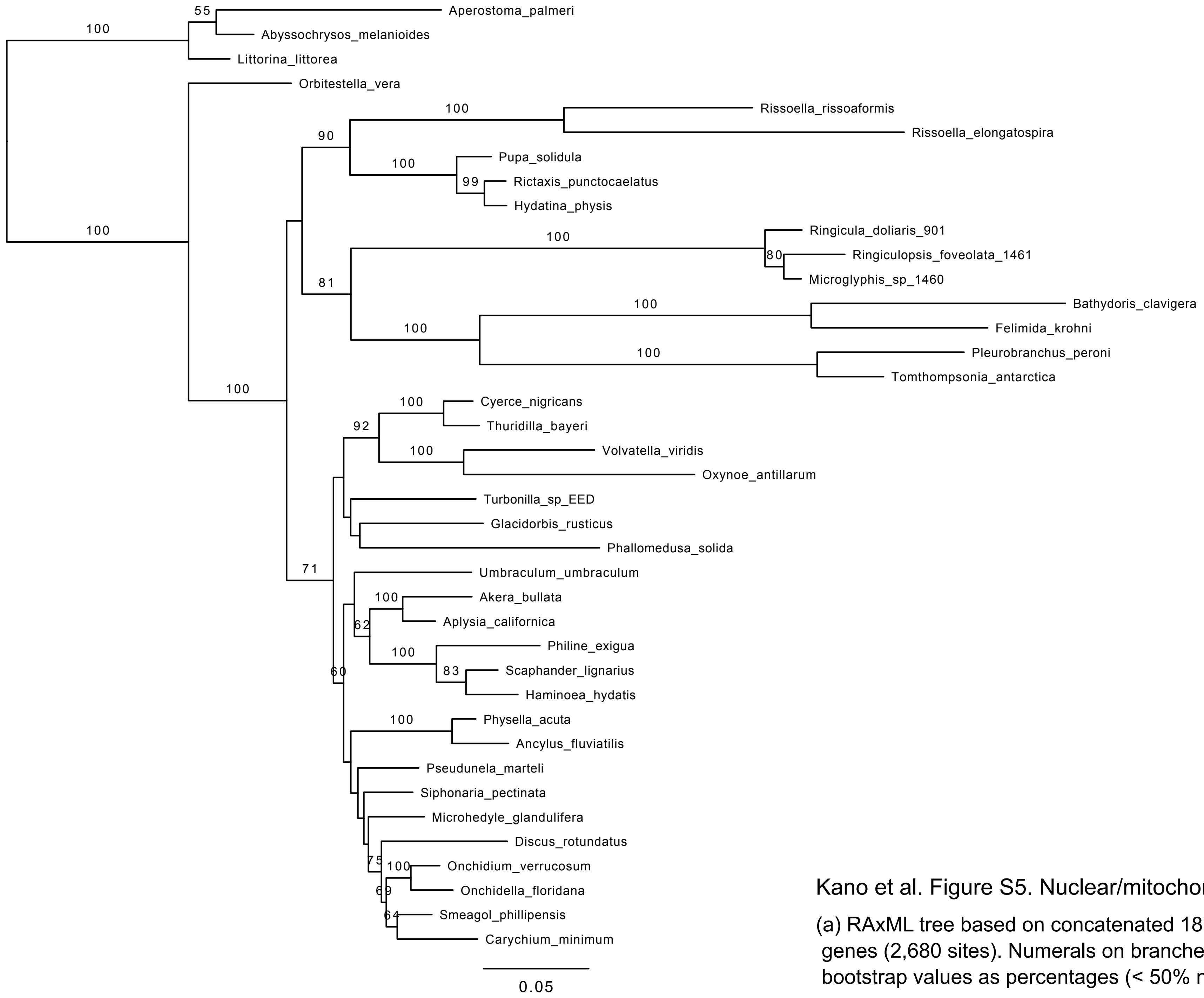

Kano et al. Figure S5. Nuclear/mitochondrial trees.

(a) RAxML tree based on concatenated 18S and 28S genes (2,680 sites). Numerals on branches denote bootstrap values as percentages (< 50% not shown).

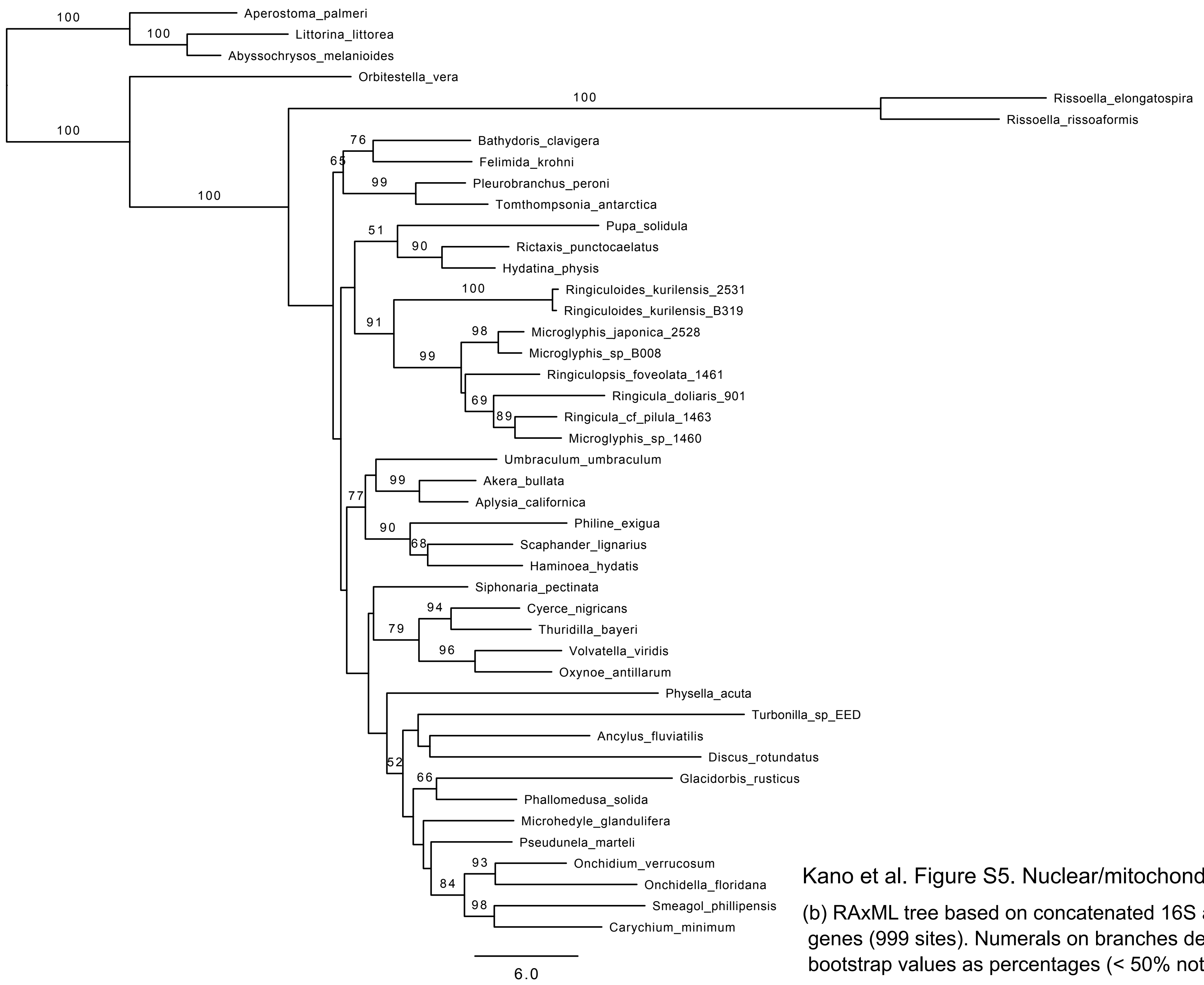

Kano et al. Figure S5. Nuclear/mitochondrial trees.

(b) RAxML tree based on concatenated 16S and COI genes (999 sites). Numerals on branches denote bootstrap values as percentages (< 50% not shown).
